# Sleep-dependent clearance of brain lipids by peripheral blood cells

**DOI:** 10.1038/s41586-025-10050-w

**Published:** 2026-02-11

**Authors:** Bumsik Cho, Diane E. Youngstrom, Samantha Killiany, Camilo Guevara, Caitlin E. Randolph, Connor H. Beveridge, Pooja Saklani, Gaurav Chopra, Amita Sehgal

**Affiliations:** 1 Department of Neuroscience, Chronobiology and Sleep Institute, University of Pennsylvania Perelman School of Medicine, Philadelphia, PA USA; 2 Howard Hughes Medical Institute, University of Pennsylvania Perelman School of Medicine, Philadelphia, PA USA; 3 Department of Chemistry, Purdue University, West Lafayette, IN USA; 4 Department of Computer Science, Purdue University, West Lafayette, IN USA; 5 Purdue Institute for Integrative Neuroscience, Purdue University, West Lafayette, IN USA; 6 Purdue Institute for Drug Discovery, Purdue University, West Lafayette, IN USA; 7 Purdue Center for Cancer Research, Purdue University, West Lafayette, IN USA; 8 Purdue Institute of Inflammation, Immunology and Infectious Disease, Purdue University, West Lafayette, IN USA

**Keywords:** Cellular neuroscience, Molecular neuroscience

## Abstract

Sleep is viewed typically through a brain-centric lens, with little known about the role of the periphery^1,2^. Here we identify a sleep function for peripheral macrophage-like cells (haemocytes) in the *Drosophila* circulation, showing that haemocytes track to the brain during sleep and take up lipids accumulated in cortex glia due to wake-associated oxidative damage. Through a screen of phagocytic receptors expressed in haemocytes, we discovered that knockdown of *eater*—a member of the Nimrod receptor family—reduces sleep. Loss of *eater* also disrupts haemocyte localization to the brain and lipid uptake, which results in increased brain levels of acetyl-CoA and acetylated proteins, including mitochondrial proteins PGC1α and DRP1. Dysregulation of mitochondria, reflected in high oxidation and reduced NAD^+^, is accompanied by impaired memory and lifespan. Thus, peripheral blood cells, which we suggest are precursors of mammalian microglia, perform a daily function of sleep to maintain brain function and fitness.

## Abstract (web summary)

Peripheral macrophage-like haemocytes in *Drosophila* promote sleep by clearing lipid buildup in the brain, helping to maintain metabolic homeostasis and brain function and fitness.

## Main

Sleep is a behavioural state shared by almost all animals. It is defined as a quiescent state associated with reduced consciousness that is different from coma or anaesthesia because it is rapidly reversible with a stimulus. The circadian system regulates sleep on a 24-h cycle, but sleep is also regulated by homeostatic mechanisms, whereby the pressure to sleep increases with extended periods of wakefulness^1^. The importance of sleep is recognized widely, but the underlying mechanisms and functions are still debated^1,2^.

Whereas studies of sleep focus on the brain, sleep loss also has an effect on the periphery^3^. In addition, there is now reason to believe that peripheral tissues can affect sleep^4,5^. For instance, the immune system is implicated in the control of sleep, particularly during sickness. In *Drosophila*, the nuclear factor kappa B protein Relish acts in the fly fat body (functional equivalent of the liver) to regulate sleep following infection^6^. Sleep, in turn, influences recovery from bacterial and viral infections in both mammals and flies^7,8^. Sleep deprivation increases the expression of sleep-promoting cytokines, such as tumour necrosis factor (TNF) or interleukin (IL)-6 and it may do so in the same way as inflammation, by increasing levels of the glucocorticoid hormone through the hypothalamic–pituitary–adrenal axis or noradrenaline through the sympathetic nervous system^7^. TNF is also implicated in *Drosophila* sleep through its expression in astrocytes, but it can act systemically as well^9^. In general, interactions between sleep and the immune system have focused largely on signalling molecules, and not on regulation by immune cells in the periphery. Little is known about the role of peripheral mechanisms in the function of sleep.

Using *Drosophila* as a model system, we addressed a sleep function for circulating blood cells called haemocytes, 95% of which are macrophage-like plasmatocytes^10^ that function in immune responses. We show that, at times of high sleep, haemocytes localize to the brain and take up lipids accumulated in cortex glia. As lipid droplets (LDs) in cortex glia reflect the transfer of wake-associated oxidative damage from neurons^11^, this uptake by haemocytes is expected to ease metabolic stress in the brain. Indeed, loss of the Eater receptor, which mediates lipid uptake by haemocytes, causes increased acetylation in the brain, along with mitochondrial oxidation and reduced NAD^+^ levels. Thus, haemocytes, and Eater in particular, act in a sleep-dependent fashion to maintain metabolic homeostasis in the brain.

## Haemocytes interact with the BBB during high sleep

**Fig. 1:**
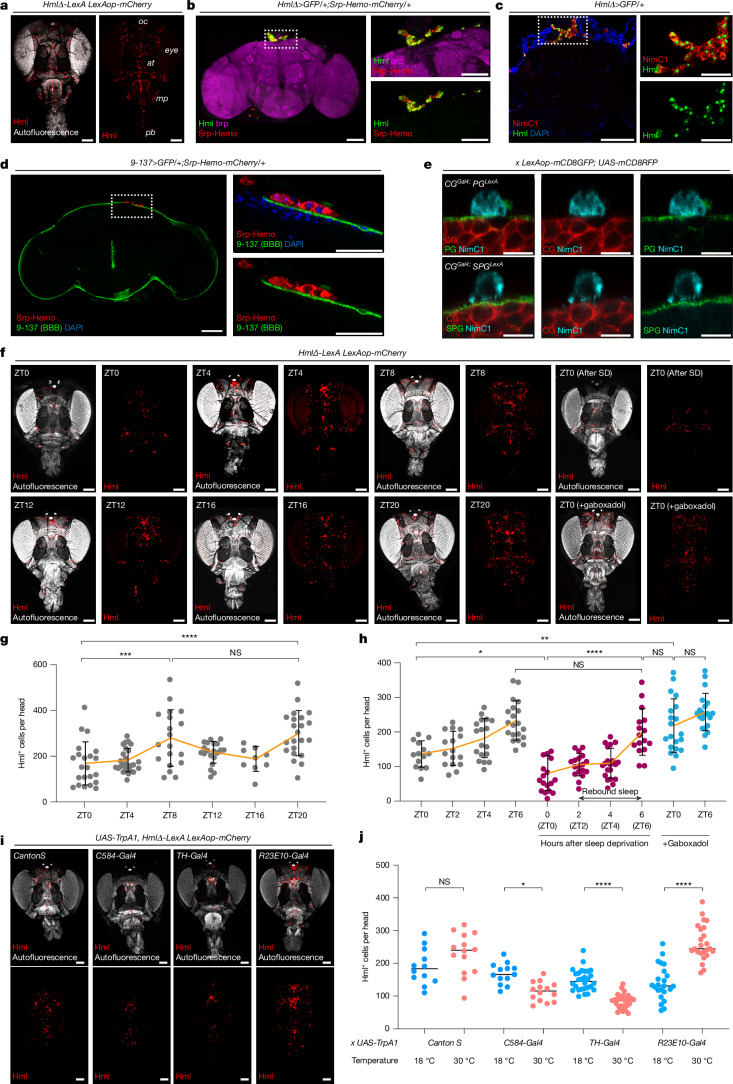
Blood cells (haemocytes) circulate in the fly head cavity and contact the BBB. **a,** Haemocytes within the fly head cavity, visualized with mCherry (red) driven by an Hml^+^ driver. **b**,**c**, Haemocytes labelled with GFP driven by Hml^+^ (green) and Srp-hemo^+^ (red) localize to the dorsal middle area of the brain (left). Dotted box shows magnified area. Brain is visualized with brp (magenta) (**b**). Hml^+^ (green) and NimC1^+^ (red) haemocytes are in the same area as Srp^+^ cells (left) (**c**). **d**,**e**, Localization of haemocytes (red) near the BBB (green) (**d**). NimC1^+^ (cyan) haemocytes are next to PG cells (*R85G01-LexA*, green) (top) or SPG glial cell (*R54C07-LexA*, green) (bottom). Cortex glia (CG) are visualized with RFP (*NP2222-Gal4*, red) (**e**). **f**–**h**, Sleep dependence of haemocyte recruitment to the head. Haemocytes (red) are visualized at different ZT times, with sleep deprivation or gaboxadol feeding (**f**). Haemocytes were quantified at different times of day (*n* = 22, 26, 19, 20, 9 and 22 from left to right on the graph, **g**) or after sleep deprivation (SD) or gaboxadol (*n* = 14, 16, 18, 20, 17, 18, 20, 16, 20 and 21 from left to right, **h**). Yellow line, average; error bars, s.d. **i**–**j**, Effects on haemocyte recruitment of manipulating sleep-promoting and wake-promoting neurons. Haemocytes (red) were visualized following wake- or sleep-promoting manipulations (**i**). Data in **i** were quantified (*n* = 12, 14, 13, 14, 28, 28, 23 and 24 from left to right on the graph; horizontal bars, median) (**j**). NS, not significant; *P* > 0.01); *P < 0.1; **P < 0.01; ***P < 0.001; ****P < 0.0001. *n* represents biologically independent samples. Two-sided Tukey’s multiple comparisons test was performed for all data analysis. Detailed statistics in Supplementary Table 1. Scale bars, 100 μm (**a**, **b**–**d** (left panels) **f**, **i**); 50 μm (**b**–**d**, right panels); 10 μm (**e**). oc, ocelli; at, antenna; mp, maxillary palp; pb, proboscis.

**Extended Data Fig. 1:**
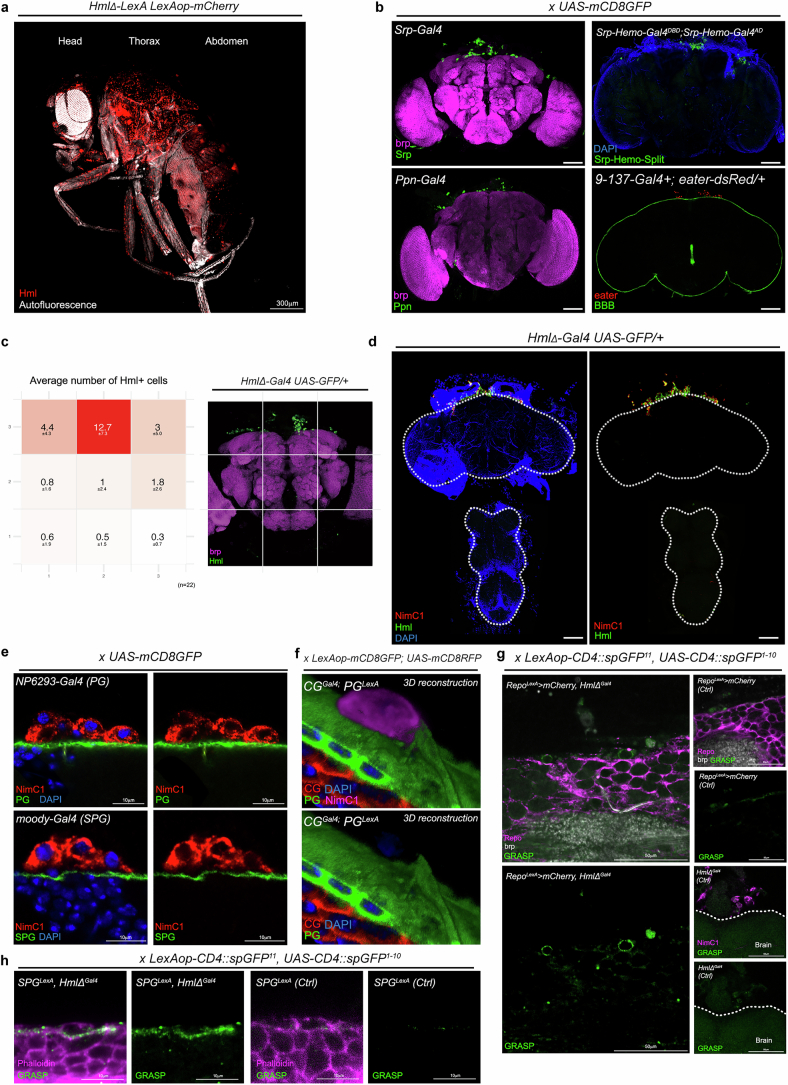
Blood cells (hemocytes) circulate in the fly head cavity. **a**. Localization of *HmlΔ-LexA*+ hemocytes (*Hml Δ-LexA LexAop-mCherry*, red) in the fly. Structure of the fly was visualized with a UV laser (Autofluorescence, white). **b-f**. Localization of the hemocytes near the brain. Visualizing of hemocyte with *Srp-Gal4* (Top left, green), *Srp-hemo-Gal4*^*DBD*^*/*^*AD*^ (Top right, green), *Ppn-Gal4* (Bottom left, green), or *eated-dsRed* (Bottom right, red). Brain was visualized with brp (magenta), DAPI (Blue). BBB was visualized with *9-137-Gal4* (bottom right, green) (**b**). Hemocytes (Hml; green, NimC1; red) are not detected in the ventral nerve cord (**c**). Heatmap of average Hml+ cell number across the brain (mean with standard deviation, n = 22) (left). Representative image of Hml+ cells (green) across the brain (brp, magenta) used for analysis (right) (**d**). NimC1+ hemocytes (red) are located next to the Perineurial glia (PG, green, *NP6293-Gal4 UAS-mCD8GFP*, top) or Sub-perineurial glia (SPG, green, *moody-Gal4 UAS-mCD8GFP*, bottom) (**e**). 3D reconstruction of Fig. 1e shows that NimC1+ (magenta) hemocytes physically interact with perineurial glial cells (green) (**f**). Cortex glial cells were visualized with RFP (red) (*R85G01-LexA LexAop-mCD8GFP NP2222-Gal4 UAS-mCD8RFP*). **g-h**. Strong GRASP signals (green) were detected between Repo+ glial cells (magenta) and hemocytes, using *Repo-LexA* and *HmlΔ-Gal4* to drive the two halves of GFP (top). In contrast, little green signal was observed in the *Repo-LexA* GRASP control samples (top right) or *HmlΔ-Gal4* GRASP control samples (bottom right). Neuropil was visualized using brp (white) (**g**). With the use of *SPG-LexA* (*R54C07-LexA)* and *HmlΔ-Gal4*, strong GRASP signals (green; left) were detected but not in *SPG-LexA* GRASP control samples (right). Cell membrane was visualized using phalloidin (magenta) (**h**). To detect *SPG-LexA* and *HmlΔ-Gal4* GRASP signals, GFP antibody was used. DAPI: blue, White scale bar, 100 μm unless it’s not indicated. All images above are representative images obtained from at least three different experiments.

To explore a possible interaction between haemocytes and the brain, we first used a tissue-clearing method^12^ to visualize circulating haemocytes in the fly head (Fig. 1a and Extended Data Fig. 1a). We used the *HmlΔ-LexA* fly line to label haemocytes, and detected their localization throughout the head area including the proboscis (pb), maxillary palp (mp) and ocellar (oc) regions, but not in the eyes or antenna (at) (Fig. 1a). To determine whether these populations of haemocytes mostly circulate or actually contact the brain, we dissected fly brains and visualized haemocytes using various markers (Fig. 1b,c and Extended Data Fig. 1b–d). Consistent with previous studies, Hml^+^ haemocytes were located mostly near the dorsal part of the brain^13^, especially in the dorsally located pars intercerebralis region (Extended Data Fig. 1c). These Hml^+^ cells were also positive for other haemocyte markers^14^ such as Srp-Hemo (Fig. 1b) and NimC1 (Fig. 1c). Moreover, they were observed with other haemocyte Gal4 drivers^14^, such as *Srp-Gal4*, *Ppn-Gal4* and *Srp-Hemo-split-Gal4*, and they expressed *eater-dsRed*, which is specific to haemocytes (Extended Data Fig. 1b). Haemocytes were not detected near the ventral nerve cord or inside the brain (Extended Data Fig. 1d). Based on these observations, we conclude that haemocytes circulate in the fly head cavity and possibly interact with the brain at specific sites.

The fly brain is separated from the periphery by the blood–brain barrier (BBB)^15^. To assess whether haemocytes near the pars intercerebralis region interact with the BBB, we visualized haemocytes together with the BBB-specific Gal4 line that marks both perineurial glia (PG) and sub-perineurial glia (SPG) cells of the BBB^15^ (Fig. 1d and Extended Data Fig. 1b), or using Gal4 lines that individually mark PG (*NP6293-Gal4*) or SPG (*moody-Gal4*) cells (Fig. 1e and Extended Data Fig. 1e). We found that Srp-Hemo^+^, *eater*^+^ or NimC1^+^ haemocytes were located adjacent to the BBB (Fig. 1d,e and Extended Data Fig. 1b,e). Moreover, when we visualized haemocytes together with PG markers, we observed that extensions of the PG membrane physically contact haemocytes (Fig. 1e and Extended Data Fig. 1f). Although SPG and haemocytes are separated by PG cells, haemocyte membranes are also in contact with SPG cells (Fig. 1e). Indeed, we used the green fluorescent protein (GFP) reconstitution across synaptic partners (GRASP) technique^16^ to confirm direct physical interaction between haemocytes and SPG cells (Extended Data Fig. 1g,h). Similar direct interaction of SPG cells with haemocytes was observed in pupal stages by electron microscopy^17^. A previous study found that cortex glia cells contact or share membranes with SPG^18^ cells, so it is possible that haemocytes also directly contact cortex glia cells but we did not detect a GRASP signal (data not shown). Based on these findings, we conclude that haemocytes exist within the fly head cavity and interact physically with glial cells, particularly glia of the BBB.

We also asked whether haemocyte recruitment to the brain is influenced by the sleep:wake cycle (Fig. 1f,g). At Zeitgeber time (ZT) 8 and ZT20 (ZT0 = lights on, in circadian terms), which are times of the afternoon siesta and night-time sleep, respectively, the number of haemocytes in the head was higher than at other times of day (Fig. 1f,g). To confirm sleep-dependent haemocyte recruitment to the fly head, we compared haemocyte numbers following sleep deprivation or gaboxadol feeding to induce sleep (Fig. 1f,h). Sleep deprivation reduced haemocyte numbers in the head, but the numbers recovered during rebound sleep (Fig. 1f,h). By contrast, feeding gaboxadol increased haemocyte numbers in the fly head, with no significant differences across ZT time points (Fig. 1f,h). These results were supported by manipulations of neuronal activity to increase/decrease sleep (Fig. 1i,j). Thermogenetic stimulation of wake-promoting neurons (*C584-Gal4 UAS-TrpA1or TH-Gal4 UAS-TrpA1*)^19^ decreased haemocyte number in the fly head, whereas similar stimulation of sleep-promoting neurons (*R23E10-Gal4 UAS-TrpA1*)^20^ increased haemocyte recruitment to the head (Fig. 1i,j). From these results, we surmise that the interaction between haemocytes and the brain is increased during sleep.

## *eater* expressed in haemocytes regulates sleep

Given that haemocytes circulate in the fly head and are more abundant during sleep than wake, we hypothesized that the function of haemocytes may be relevant for sleep. To use an unbiased approach towards such function, we examined recent single-cell RNA sequencing data^21^ for transcripts expressed highly in haemocytes. Analysis of the biological functions of the top 100 genes in haemocytes through g:Profiler^22^ revealed significant annotations for defence response against other organisms, phagocytosis and immune system processes. As the phagocytosis of Gram-positive or Gram-negative bacteria in *Drosophila* is mediated typically by Nimrod receptor family genes^23^, we focused on a possible role for this family (*NimA*, *NimB1-5*, *NimC1-4*, *drpr*, *eater*, *Col4a1*, *PGRP-LC*) in the regulation of sleep. In addition, because we observed that more haemocytes are localized in the head cavity during the sleep state in flies (Fig. 1f–j), we also added genes previously identified as being involved in haemocyte migration. These include activin-β signalling factors (*babo*, *put*, *Smox*) that are important for sessile localization of haemocytes^24^, or the platelet-derived growth factor/vascular endothelial growth factor signalling receptor that functions in embryonic blood cell migration^25^. Finally, we included genes associated with lipid uptake (*Lsd-1*, *Lsd-2*, *GLaz*, *Karl*, *Nplp2*, *Apoltp*, *Acsl*, *LpR1*, *LpR2*, *eater*, *crq*, *apolpp*), as previous studies have shown that haemocyte functions are linked to lipid uptake^26^, processing^27^ and clearance^28^, which are critical for immune system activation, animal growth and metabolism.

**Fig. 2:**
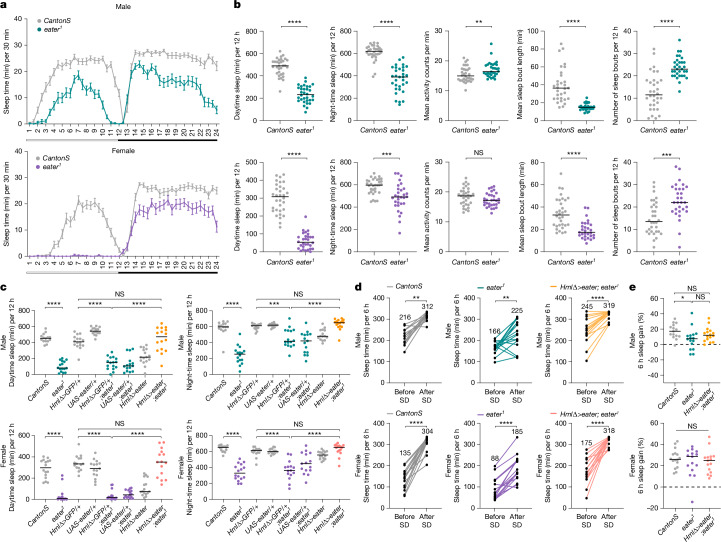
*eater* in haemocytes regulates sleep. **a**,**b**, Analysis of sleep in wild-type (*CantonS*) and *eater* mutant (*eater*^*1*^) flies. Graphs indicate median (± s.e.m.) sleep amount in flies (**a**). White and black bars below graph represent day and night, respectively. Daytime sleep, night-time sleep, mean activity counts per minute, mean sleep bout length and number of night-time sleep bouts in male (top, dark green) and female (bottom, purple) flies were quantified (**b**). Two-sided Mann–Whitney test was performed for data analysis; *n* = 32, 32, 32 and 30 for *CantonS* male, female, *eater*^*1*^ male and female, respectively. **c**–**e**, Rescue of sleep reduction in *eater* mutants through haemocyte-specific *eater* expression. Sleep time was quantified in *eater* mutants (**c**) (green and purple dots) and following *eater* expression in mutants (yellow and pink dots) (*n* = 15, 16, 15, 16, 16, 15, 15 and 16 from left to right on the graph, male) (*n* = 16, 16, 15, 15, 15, 15, 16 and 15 from left to right, female). Sleep time was assessed during and after sleep deprivation in male and female flies (**d**). Numbers above the each array of dots represent the total sleep time (in minutes) over 6 h for each genotype; *n* = 15, 16, 16, 15, 16 and 15 for *CantonS* male, female, *eater*^*1*^ male, female and *HmlΔ>eater; eater*^*1*^ male and female, respectively. Rebound sleep was quantified as gain in sleep ZT0–6 after sleep deprivation relative to the same period before sleep deprivation (dark green and purple) (**e**). Two-sided Tukey’s multiple comparisons test was performed for data analysis in **c** and **e**. Two-sided paired *t*-test was performed for data analysis in **d**. NS, *P* > 0.01; *P < 0.1; **P < 0.01, ***P < 0.001, ****P < 0.0001. *n* represents biologically independent samples. Detailed statistics in Supplementary Table 1.

**Extended Data Fig. 2:**
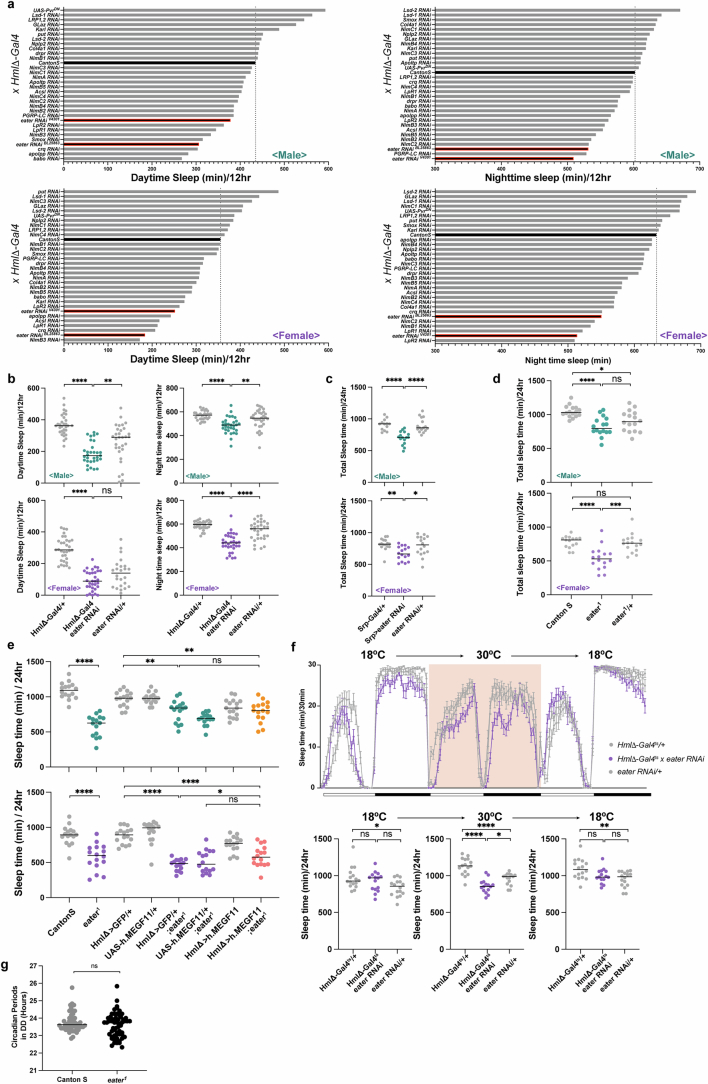
*eater* is required in hemocytes to regulate sleep. **a**. Screen for genes regulating sleep in hemocytes using RNAi knockdown with *HmlΔ-Gal4*. The sleep amount in wild-type flies is indicated by a black column with a dotted line. Knockdown of *eater* using two different RNAi lines resulted in decreased daytime or nighttime sleep in male and female (columns with red lines). **b**. Graphs represent sleep in wild-type (*CantonS*) and *eater* knockdown flies (*HmlΔ-Gal4 UAS-GFP*, *eater RNAi*) for both males (top, dark green) and females (bottom, purple). Daytime and nighttime sleep are reduced in males (top, dark green) and mostly night-time in females (bottom, purple). *n* = 31, 31, and 32 for male and *n* = 32, 32, and 31 for female. From left to right on the graph. Two-sided Tukey’s multiple comparisons test was performed for data analysis. **c**. Quantification of sleep time in *eater* knockdown using a different hemocyte driver (*Srp-Gal4*). Sleep is shown in males (top) and females (bottom). *n* = 13, 16, and 16 for male and *n* = 16, 16, and 16 for female. From left to right on the graph. Two-sided Tukey’s multiple comparisons test was performed for data analysis. **d**. Quantification of sleep in homozygous or heterozygous *eater* mutant flies (top, dark green, male) (bottom, purple, female). *n* = 16, 16, and 16 for male and *n* = 15, 16, and 16 for female. From left to right on the graph. Two-sided Tukey’s multiple comparisons test was performed for data analysis. **e**. Hemocyte specific overexpression of *h.MEGF11* in *eater* (*eater*^*1*^) mutant background. Graphs represents total sleep time. Dark green dots represent *eater* mutant males. Yellow dots represent hemocyte specific *h.MEGF11* overexpression in the *eater* mutant males (top). Purple dots represent *eater* mutant female. Pink dots represent hemocyte specific *h.MEGF11* overexpression in the *eater* mutant female (bottom). *n* = 16, 15, 16, 16, 16, 16, 16 and 16 for male and *n* = 16, 16, 16, 16, 16, 16, 16 and 16 for female. From left to right on the graph. Two-sided Tukey’s multiple comparisons test was performed for data analysis. **f**. Comparison of sleep time in *eater* knock-down flies only in the adult stage using the temperature sensitive Gal80 (*HmlΔ-Gal4 UAS-GFP*, *Tub-Gal80*^*ts*^, *eater RNAi*). Graph on the top left side represents total sleep time before Gal4 activation. Graph in the top middle represents total sleep time during Gal4 activation. Graph on the top right side represents total sleep time following return to de-activating temperature. Red shade indicates Gal4 activating time. Quantification on the bottom. *n* = 16, 15, and 16. Bars in graphs: the median with SEM. White and black bar below the graph represents day (white) and night (black). Two-sided Tukey’s multiple comparisons test was performed for data analysis. **g**. Quantification of circadian periods in wild-type (*CantonS*) or *eater* mutant (*eater*^*1*^) in dark-dark condition. *n* = 50 for *CantonS* and *n* = 58 for *eater*^*1*^. Two-sided Mann-Whitney test was performed for data analysis. ns: not significant (p > 0.01); *p < 0.1; **p < 0.01, ***p < 0.001. ****p < 0.0001. Bars in graphs: the median. *n* represents biologically independent samples.

We then knocked down each of the genes above using RNA interference (RNAi) and assayed effects on sleep. Knockdown of the gene *eater* reduced sleep, as validated using two different *eater* RNAi lines (Extended Data Fig. 2a) with two distinct haemocyte Gal4 drivers (Extended Data Fig. 2b,c). To further confirm the *eater* knockdown phenotype, we assessed the sleep patterns of *eater* null mutants. Both male and female *eater* mutant flies exhibited reduced daytime and night-time sleep (Fig. 2a,b), with shorter sleep bout lengths and more sleep bouts during the night-time but no reduction in activity counts during waking time (Fig. 2b). This phenotype indicates that the reduced sleep of the *eater* mutants is also fragmented and not associated with motor activity impairment. Reduction of sleep was not observed in *eater* heterozygous flies (Extended Data Fig. 2d), indicating that the effect is recessive. Transgenic expression of *eater* in haemocytes rescued the mutant sleep phenotype, confirming that the phenotype maps to the *eater* gene (Fig. 2c). We attempted to also rescue *eater* with a homologous mammalian protein, MEGF11 (ref. ^29^), but this was not successful (Extended Data Fig. 2e).

To exclude the possibility that the reduced sleep phenotype comes from developmental effects, we used a temperature-sensitive Gal80 (*HmlΔ-Gal4 UAS-GFP, Tub-Gal80ts*) to block Gal4 expression during development. Knocking down *eater* in the adult stage only was enough to decrease sleep, indicating that the phenotype is not due to developmental effects (Extended Data Fig. 2f). Furthermore, lack of a circadian phenotype under constant dark conditions (Extended Data Fig. 2g) demonstrated that the decreased sleep amount in *eater* mutants is not driven by circadian rhythm alterations, indicating that *eater* affects sleep more directly.

To assess whether *eater* mutants exhibit rebound sleep, flies were subjected to sleep deprivation, and the amount of sleep gained during recovery was compared with that in control flies (Fig. 2d,e). Although the total sleep during a 6-h recovery period was lower in *eater* mutants than in controls, the percentage of sleep gain was comparable across groups (Fig. 2d,e). These results indicate that homeostatic regulation of sleep is intact in *eater* mutants. Altogether, we conclude that *eater* in haemocytes is required to maintain daily sleep in adult flies.

## Haemocyte transfer rescues sleep in *eater* mutants

**Extended Data Fig. 3:**
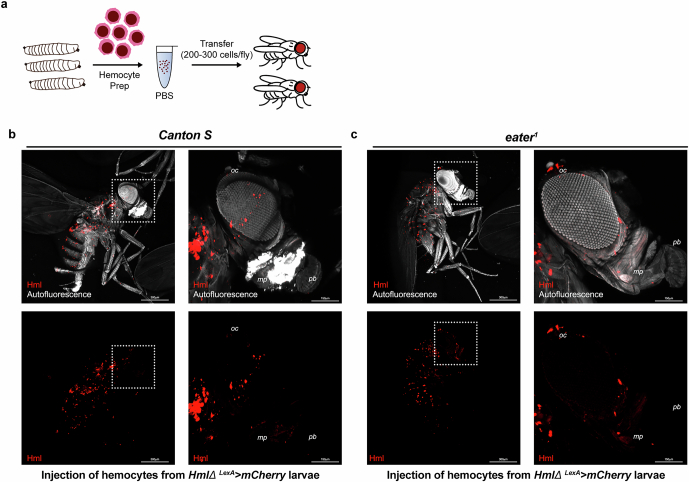
Hemocyte transfer rescues the *eater* mutant sleep phenotype. **a**. Schematic representation of hemocyte transfer experiment. Images were generated by Illustrator. **b-c**. Confirmation of larval hemocyte transfer to the adult fly in wild-type (**b**) and *eater* mutants (**c**). *Hml-LexA*+ hemocytes transferred from the larvae (*Hml Δ-LexA LexAop-mCherry*, red) were visualized in the fly. Structure of the fly was visualized with UV laser (Autoflourescence, white). Magnified images from the dotted box are on the right side.

Although *eater* is known to be expressed specifically in haemocytes^30^, we further tested whether the mutant sleep phenotype derives from haemocytes by transferring wild-type haemocytes to *eater* mutant flies to determine whether this could restore their normal sleep pattern. Due to the challenge of obtaining pure haemocytes from adult flies without any enzymatic treatment, we used larval haemocytes for the transfer experiment (Extended Data Fig. 3a). Using the tissue-clearing method, we confirmed that transferred labelled haemocytes were circulating properly throughout the body and head regions of wild-type and *eater* mutant flies 4 h after injection (Extended Data Fig. 3b,c).

**Fig. 3:**
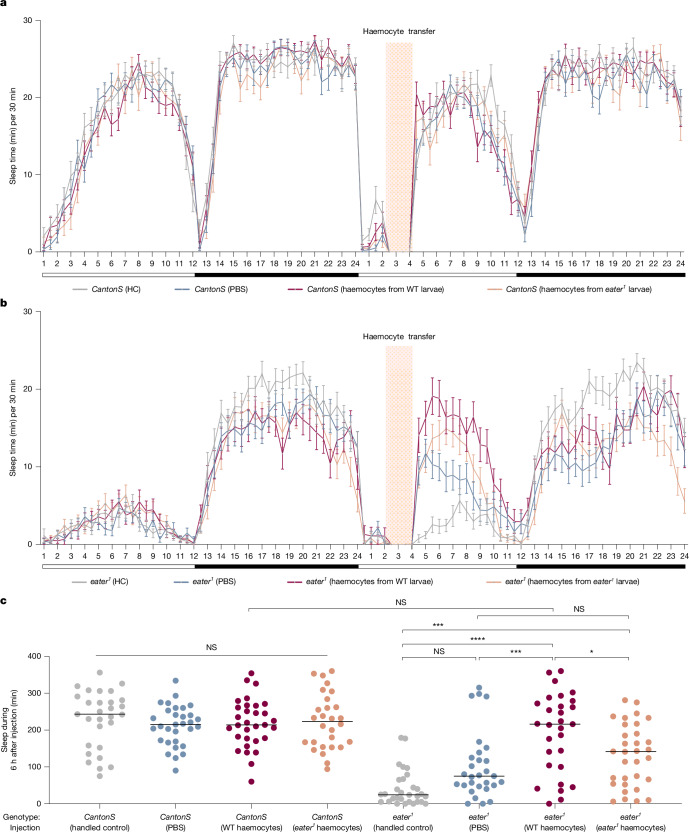
Haemocyte transfer rescues the *eater* mutant sleep phenotype. **a**–**c**, Comparison of sleep amount in wild-type (*CantonS*) and *eater*^*1*^ before and after larval haemocyte transfer. Sleep was profiled in *CantonS* (**a**) and *eater*^*1*^ (**b**). Sleep was quantified during the 6 h after haemocyte transfer in both wild-type and *eater* mutant flies (**c**). HC, hand control (grey; not wounded but exposed to CO_2_); PBS, PBS injection (blue); WT, wild type (dark red; *CantonS* haemocyte transfer); *eater*^*1*^, *eater*^*1*^ haemocyte transfer (orange). Two-sided Tukey’s multiple comparisons test was performed for data analysis; *n* = 29, 31, 31, 28, 30, 29, 29 and 32 from left to right on the graph. NS, *P* > 0.01; *P < 0.1; **P < 0.01, ***P < 0.001, ****P < 0.0001. Horizontal bars in graphs: the mean with s.e.m. White and black bars below the graphs in **a** and **b** represent day and night, respectively. The red shading in **a** and **b** indicates time point of haemocyte transfer. *n* represents biologically independent samples. Detailed statistics in Supplementary Table 1.

We then assessed whether the haemocyte transfer rescued the sleep phenotype in *eater* mutants. We injected haemocytes at the beginning of the day, ZT2, to minimize wound-induced increases in sleep that occur with injection at night^6^. In wild-type flies, transfer of either wild-type or *eater* mutant haemocytes did not alter sleep amount or patterns (Fig. 3a,c). In *eater* mutants, injection alone (for example, with phosphate-buffered saline (PBS)) elicited a small, but insignificant, increase in sleep. However, injection with wild-type haemocytes produced the most robust sleep increase—significantly higher than in flies injected with PBS or *eater* mutant haemocytes (Fig. 3b,c). The fact that both wild-type haemocyte transfer to *eater* mutants (Fig. 3b,c) and genetic restoration of *eater* expression in the haemocytes of *eater* mutants (Fig. 2c) are able to rescue the sleep loss phenotype confirms that *eater* function in haemocytes is necessary and sufficient to regulate sleep.

## Haemocyte Eater clears lipids from cortex glia

The Eater protein, which contains 32 epidermal growth factor (EGF)-like repeats, is known to be involved in three key functions: (1) phagocytosis of Gram-positive bacteria^31^, (2) cell-to-cell adhesion^32^ and (3) low-density lipoprotein (LDL) uptake^30^. These functions are also conserved in mammalian proteins containing EGF-like repeats^33^. Because we did not deliver bacterial challenges to the fly, we investigated the other two functions of Eater.

**Fig. 4:**
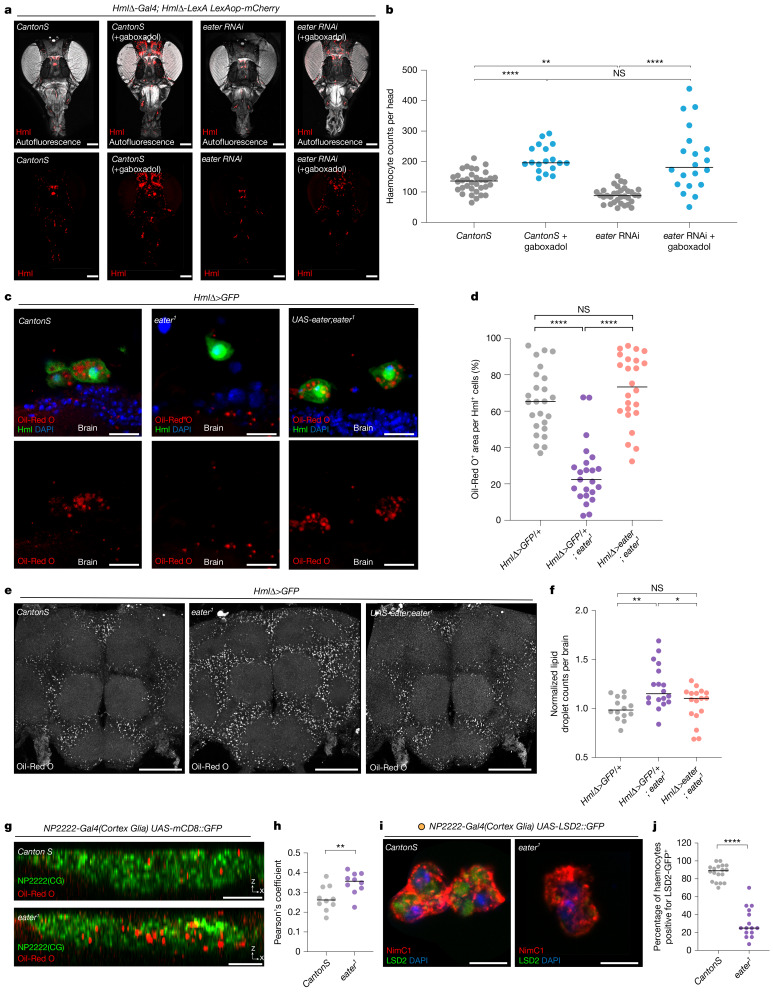
Eater is required for haemocytes to take up lipids from cortex glial cells. **a**,**b**, Effect of increasing sleep on head recruitment of haemocytes in flies with reduced *eater*. Wild-type or *eater* knockdown haemocytes (red) are visualized at ZT2 or after gaboxadol feeding (**a**). Haemocytes in **a** were quantified (**b**); *n* = 34, 18, 33 and 20 from left to right. **c**,**d**, LDs in haemocytes of wild-type, *eater* mutant and *eater* rescue backgrounds. Visualization was with Oil-Red O (red) (**c**). Oil-Red O areas in **c** were quantified (**d**); *n* = 25, 23, and 24 from left to right. **e**–**h**, LDs in brains of wild-type, *eater* mutant and *eater* rescue backgrounds; *eater* mutants have more LDs (grey) (**e**). Oil-Red O areas from **e** were quantified (**f**); *n* = 14, 18 and 17 from left to right on the graph. A cortex glia marker was used to visualize LDs (red) in these cells in wild-type (top) or *eater* mutants (bottom) (**g**). Pearson’s coefficient was calculated for Oil-Red O co-localization with cortex glia marker (**h**); *n* = 10 of each. **i**,**j**, Cortex-glia-derived LDs in haemocytes. LSD2::GFP^+^ LDs from cortex glia (green) were detected in NimC1 positive (red) haemocytes. Control haemocytes (left) contain more LDs than those from *eater* mutants (right) (**i**). The LSD2::GFP^+^ area within individual haemocytes was quantified (**j**); *n* = 18 for *CantonS* and 15 for *eater*^*1*^. Two-sided Tukey’s multiple comparisons test was performed for **b**, **d** and **f** and two-sided unpaired *t*-test for **h**. Two-sided Mann–Whitney test was performed for **j**. NS, *P* > 0.01; *P < 0.1; **P < 0.01, ***P < 0.001, ****P < 0.0001. Horizontal bars in graphs, median. *n* represents biologically independent samples. Detailed statistics in Supplementary Table 1. Scale bars, 100 μm (**a**, **e**); 10 μm (**c**, **i**); 20 μm (**g**).

**Extended Data Fig. 4:**
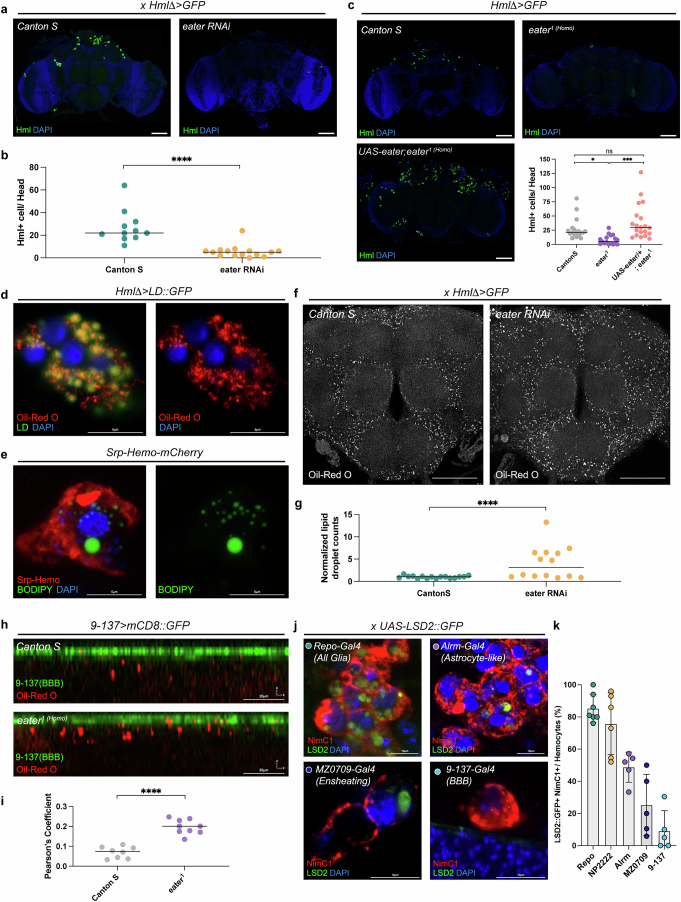
Hemocytes take up lipids from cortex glia cells through *eater.* **a-b**. Detection of Hml+ (green) hemocytes within wild-type (*HmlΔ>GFP/+*) and *eater* knockdown (*HmlΔ>GFP, eater RNAi*) flies (**a**). Quantification of Hml+ hemocytes near the brain showed reduction in *eater* mutants. *n* = 11 for *CantonS*, *n* = 11 for *eater*^*1*^ (**b**). Two-sided Mann-Whitney test was performed for data analysis. **c**. Detection of Hml+ (green) hemocytes within wild-type (*HmlΔ>GFP/+*; top left), *eater* mutant (*HmlΔ>GFP/+, eater*^*1*^; top right*)* and *eater* rescue background (*HmlΔ>GFP, UAS-eater/+, eater*^*1*^; bottom left). *n* = 18, 17, and 20. From left to right on the graph. Quantification of hemocytes is on the bottom right. Two-sided Tukey’s multiple comparisons test was performed for data analysis. **d-e**. Detection of lipid droplets in hemocytes. Oil-Red O lipid staining in hemocytes (red) localized to lipid droplets labeled with GFP (green, *HmlΔ-Gal4/UAS-LD::GFP*) (**d**). BODIPY (green) positive lipid droplet in the Srp-hemo+ (red) hemocyte (**e**). **f-g**. Detection of lipid droplets in the brain with Oil-Red O (gray) in wild-type (*HmlΔ>GFP/+*) and *eater* knockdown (*HmlΔ>GFP, eater RNAi*) (**f**). Quantification of lipid droplets is shown in (**g**). *n* = 16 for *CantonS*, *n* = 14 for *eater RNAi*. Two-sided Mann-Whitney test was performed for data analysis. **h-i** Detection of Oil-Red O positive lipid droplets (red) in the brain with a blood-brain barrier marker in wild-type (top, *9-137-Gal4 UAS-mCD8GFP/+*) or *eater* mutant (bottom, *9-137-Gal4 UAS-mCD8GFP/+, eater*^*1*^) (**h**). Pearson’s coefficient of Oil-Red O co-localization with BBB glial cell marker (**i**). *n* = 8 for *CantonS*, *n* = 9 for *eater RNAi*. Two-sided Unpaired t test was performed for data analysis in (i). **j-k**. Detection of lipid droplets derived from different glial cells in hemocytes. NimC1+ (red) hemocytes were co-localized with LSD2::GFP positive lipid droplets originating from all glia (*Repo-Gal4 UAS-LSD2::GFP)*, astrocyte-like glia (*Alrm-Gal4 UAS-LSD2::GFP*), ensheathing glia (*MZ0709-Gal4 UAS-LSD2::GFP*), and BBB glia (*9-137-Gal4 UAS-LSD2::GFP*) (**j**). Quantification of LSD2::GFP+ hemocytes is in (**k**). *n* = 7, 6, 5, 5 and 5. From left to right on the graph. each dot represents biologically independent experiments. ns: not significant (p > 0.01); *p < 0.1; **p < 0.01, ***p < 0.001. ****p < 0.0001. Bars in graphs: the median with SD (k). DAPI: blue, White scale bar, 100 μm unless otherwise indicated. *n* represents biologically independent samples.

First, we assayed the number of circulating haemocytes in the fly head cavity in the *eater* knockdown background and found that the number was reduced relative to wild type (Fig. 4a,b). The number of head haemocytes was rescued when sleep was increased by gaboxadol feeding but with high variability from fly to fly (Fig. 4a,b), which could reflect impaired localization to glial cells. Thus, we also examined whether the loss of *eater* in haemocytes affects their proximity to glial cells at the brain surface (Extended Data Fig. 4a–c). Both with *eater* knockdown and *eater* mutants, fewer haemocytes were observed near glial cells, consistent with the reduced number in the head cavity (Extended Data Fig. 4a–c), and this was rescued by re-introducing *eater* expression in haemocytes (Extended Data Fig. 4c). Given that this manipulation was also sufficient to rescue sleep, together with the brain association of haemocytes at times that correspond to sleep (Figs. 1f–j and 4a,b and Extended Data Fig. 4a–c), we conclude that haemocyte recruitment and localization to glial cells influences sleep in flies.

Next we investigated whether haemocytes take up LDs through Eater. First, we visualized LDs using a GFP-tagged LD domain^34^ in haemocytes (Extended Data Fig. 4d). We found that haemocytes were positive for Oil-Red O staining, and that this staining co-localized with LD-GFP (Extended Data Fig. 4d). Furthermore, we confirmed that lipids in the haemocytes were positive for BODIPY (Extended Data Fig. 4e). Oil-Red O staining in haemocytes was reduced significantly in *eater* mutants (Fig. 4c,d). This reduction in Oil-Red O staining in haemocytes was restored when Eater was rescued in haemocytes (Fig. 4c,d), indicating that *eater* is important for LD uptake into haemocytes.

We showed previously that LD accumulation in glial cells changes over the sleep–wake cycle and increases following sleep deprivation^11^. Given that *eater* mutants exhibit reduced sleep compared with wild-type flies and less lipid accumulation in haemocytes, we examined LD accumulation in brains (Fig. 4e,f). Compared with wild type, Oil-Red O-positive LDs were increased in glial cells of *eater* mutants (Fig. 4e,f) or with *eater* knockdown in haemocytes (Extended Data Fig. 4f–g), and their accumulation was reduced when *eater* was rescued in haemocytes (Fig. 4e,f). Consistent with a previous report^11^, we found that most LDs accumulate in cortex glia, with lower levels in the BBB (Fig. 4g,h and Extended Data Fig. 4h–i). LD accumulation in the BBB also appeared to be higher in *eater* mutants, suggesting that these cells are also affected by loss of Eater.

We next tested the hypothesis that LDs in haemocytes are derived from glia. We expressed GFP-tagged lipid storage droplet2 (LSD2) (*UAS-LSD2::GFP*)^35^ in glial cells using the pan-glial driver (*Repo-Gal4*) and checked whether LSD2::GFP was transferred to haemocytes. More than 80% of haemocytes displayed a glial cell derived LSD2::GFP signal, demonstrating that haemocytes take up LDs from glial cells (Extended Data Fig. 4j–k). To determine which glial subpopulations transfer LDs to haemocytes, we used specific glial drivers. Given that LDs are known to accumulate in cortex glia, it was not surprising to find that approximately 75% of haemocytes were LSD2::GFP-positive when using a cortex glia driver (*NP2222-Gal4*)—a level comparable to that observed with the pan-glial driver (Extended Data Fig. 4j–k). This suggests that cortex glia are the predominant glial cells transferring LDs to haemocytes. By contrast, when using drivers specific for other glial subpopulations, approximately 50% of haemocytes were LSD2::GFP-positive with an astrocyte-like glia driver (*Alrm-Gal4*), around 20% with an ensheathing glia driver (*MZ0709-Gal4*) and about 10% with a BBB glia driver (*9-137-Gal4*) (Extended Data Fig. 4j–k). When LSD2::GFP was expressed with the cortex glia driver (*NP2222-Gal4*) in the *eater* mutant background, fewer LSD2::GFP droplets were observed in haemocytes (Fig. 4i,j). Overall, we conclude that haemocytes interact with glial cells, particularly cortex glia, to uptake LDs through the Eater protein. If the cell-adhesion or LD-uptake function of Eater is diminished, then excess lipids accumulate in cortex glia.

## Eater mediates uptake of acetylated lipoproteins

**Extended Data Fig. 5:**
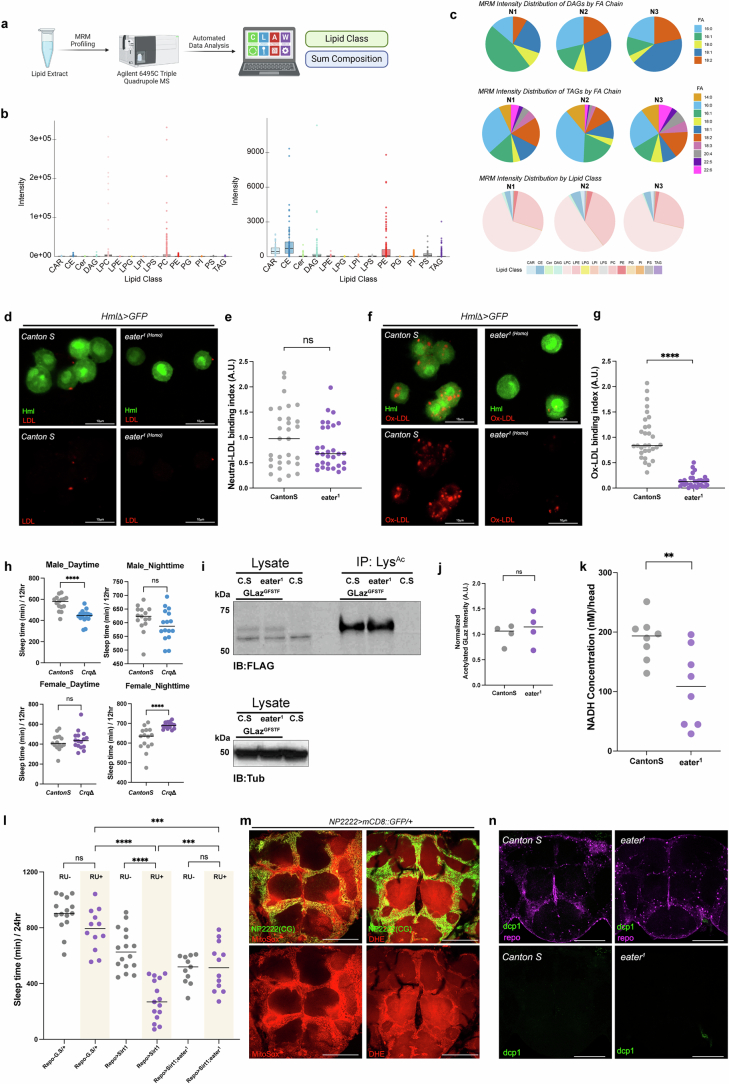
Protein acetylation is increased in the *eater* mutant brain. **a-c**. Analysis of lipid classes in hemocytes. A lipidomic analysis pipeline utilizing Multiple Reaction Monitoring (MRM) profiling with an Agilent 6495 C Triple Quadrupole Mass Spectrometer (MS) followed by automated data analysis. The data are depicted as a boxplot representation of the intensity distribution of various lipid classes detected in wild-type hemocyte samples (*HmlΔ>GFP/* +, N = 3) using multiple reaction monitoring (MRM)-based lipidomics. The full intensity distribution across lipid classes, highlighting variations in signal intensities, is shown (left). On the right is a zoomed-in view providing a more detailed comparison of lipid classes with lower intensity signals (right). Each lipid class is color-coded, and individual data points represent detected lipid species (**b**). Diacylglycerols (DAGs), triacylglycerols (TAGs), and lipid classes in hemocytes are depicted by MRM intensity distribution. Pie charts represent the relative intensity distribution of DAG species categorized by fatty acid (FA) chain composition across three biological replicates (N1, N2, N3) (top). The relative intensity distribution of TAG species is indicated by FA chain composition, showcasing the diversity of FA chains (middle). Predominant lipid classes detected are indicated by lipid class distribution within the lipidomic profile (bottom). The color legends correspond to the FA chain composition and lipid classes in each panel (**c**). The box represents the interquartile range (25%–75% percentiles) and the line inside box means median. **d-g**. Ex vivo culture of hemocytes with neutral or oxidized LDL. Neutral LDL (red) were barely observed within or attached to wild-type (left, green, *HmlΔ>GFP/+, n* = 30) or *eater* mutant (right, green, *HmlΔ>GFP/+, eater*^*1*^*, n* = 30) hemocytes (**d**). Quantification of the bound neutral LDL on the hemocytes. Bounded amount was normalized to *CantonS* hemocytes (**e**). Oxidized LDL (OxLDL) (red) were observed inside the wild-type hemocytes (left, green, *HmlΔ>GFP/+, n* = 30) and *eater* mutants (right, green, *HmlΔ>GFP/+, eater*^*1*^*, n* = 30) (**f**). Quantification of the bound oxidized LDL in the hemocytes. Bounded amount was normalized to *CantonS* hemocytes (**g**). Two-sided Mann-Whitney test was performed for data analysis. **h**. Quantification of daytime and nighttime sleep time in the *crq* mutant (*CrqΔ*) fly (top; male, bottom; female). *n* = 15 for *CantonS*, *n* = 16 for *CrqΔ* males, *n* = 15 for *CantonS*, *n* = 16 for *CrqΔ* females. Two-sided Mann-Whitney test was performed for data analysis. **i-j**. Immunoprecipitation of acetylated GLaz from wild-type or *eater* mutant brain. GLaz expression level in the wild-type (*CantonS*, C.S, *n* = 4) or *eater* mutant (*eater*^*1*^*, n* = 4) is similar in the lysate. The level of acetylated-GLaz is also similar (**i**). This is supported by quantification of acetylated-GLaz levels (**j**). IB: immunoblot. IP: Immunoprecipitation. Lys^AC^: acetylated lysine. Tub: alpha tubulin. Two-sided Mann-Whitney test was performed for data analysis. *n* represents biologically independent experiments. **k**. Measurement of NADH levels in *eater* mutant (*eater*^*1*^*, n* = 8) and wild-type (*CantonS, n* = 8) heads. Two-sided Unpaired t test was performed for data analysis. *n* represents biologically independent experiments. **l**. Graphs comparing total sleep time in female wild-type (*Repo-GeneSwitch(G.S)/+*), sirtuin overexpression (*Repo-GeneSwitch(G.S) UAS-Sirt1/+*), and *eater* mutant with sirtuin overexpression (*Repo-GeneSwitch(G.S) UAS-Sirt1/+, eater*^*1*^) in glial cells. The red shade indicates the sleep data for flies fed with RU486. *n* = 15, 12, 16, 15, 11, and 12. From left to right. Two-sided Tukey’s multiple comparisons test was performed for data analysis. **m**. Visualization of ROS using fluorescence probes in the brain. ROS dyes (left, MitoSox, right, DHE) were co-localized with cortex glial cell marker (green, *NP2222-Gal4 UAS-mCD8GFP/+*). **n**. Staining of cleaved Dcp1 as a cell-death marker in the fly brain. There are no differences in Dcp1 staining (green) in wild-type (*CantonS*, left) and *eater* mutants (*eater*^*1*^, right). Glial cell was visualized by repo antibody (magenta). ns: not significant (p > 0.01); *p < 0.1; **p < 0.01, ***p < 0.001. ****p < 0.0001. Bars in graphs: the median. White scale bar, 100 μm unless otherwise indicated. *n* represents biologically independent samples except ss(j), (k). Schematic in** a** was created using BioRender (https://biorender.com).

Using a multiple reaction monitoring (MRM)-based lipidomic screening approach^36,37^, we screened lipid species in haemocytes isolated from the head cavity (Extended Data Fig. 5a–c). Phospholipids, including both diacyl and lyso-species such as phosphatidylcholines, lysophosphatidylcholines, phosphatidylethanolamines and phosphatidylserines, had the highest average intensity across all replicates (Extended Data Fig. 5b,c). Among these, lysophosphatidylcholines and phosphatidylcholines exhibited the highest individual intensities. Although these experiments are not fully quantitative, the data indicate that phospholipids are the predominant lipid components in haemocytes, consistent with previous findings^38^. By contrast, other lipid classes, including carnitines and cholesteryl esters, contributed lower signal intensities, which could reflect reduced relative abundance. Cholesteryl ester species, which are a principal component of LDL^39,40^, displayed a broad intensity distribution in head haemocytes. Probably because of the high sensitivity of MRM-screening approaches, we successfully detected cholesteryl esters in head haemocytes, whereas previous studies did not^38^. We hypothesize that cholesteryl ester results from LDL uptake by haemocytes from glia and is processed when they leave the head cavity (Extended Data Fig. 5b,c). We further focused on the LDL uptake function of Eater in haemocytes.

**Fig. 5:**
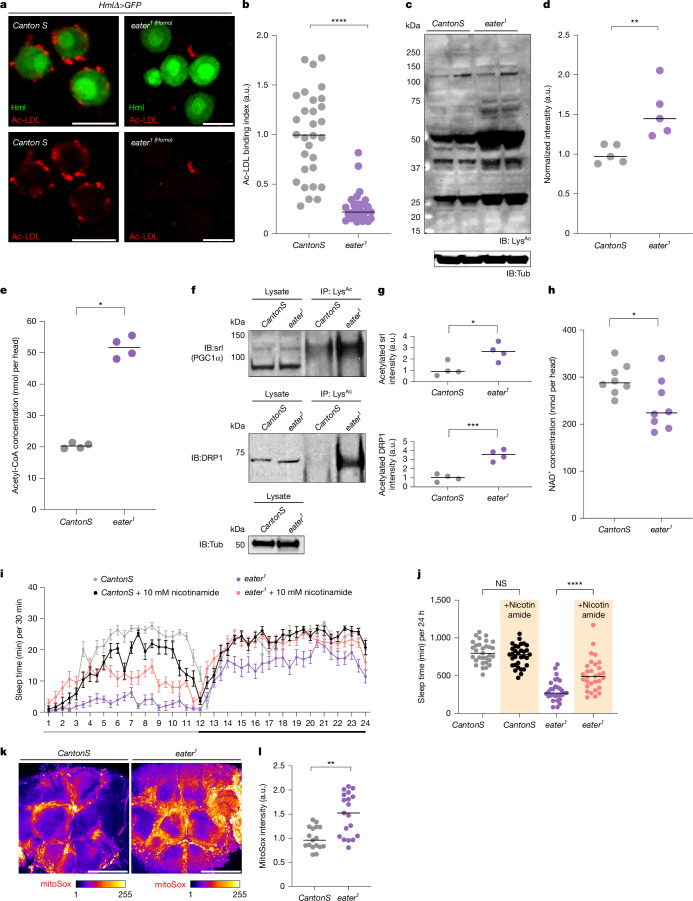
Protein acetylation is increased in *eater* mutant brains. **a**,**b**, Ex vivo culture of haemocytes with acetylated LDL (Ac-LDL). Acetylated LDL (red) was observed on the surface of wild-type (left*, n* = 30) but not *eater* mutant haemocytes (right, *n* = 30) (**a**). Bound acetylated LDL was quantified, normalized to wild-type *CantonS* haemocytes (**b**). **c**,**d**, Comparison of acetylated proteins in *CantonS* and *eater*^*1*^ fly heads. Western blot analysis of acetylated proteins (**c**). Acetylated proteins in **c** were quantified (**d**). **e**, Acetyl-CoA levels in *eater*^*1*^ and *CantonS* heads. **f**,**g**, Immunoprecipitation of acetylated proteins in wild-type or *eater* mutant heads. Acetylated proteins were immunoprecipitated and Srl (top) or DRP1 (bottom) were detected by western blot in *CantonS* or *eater*^*1*^ (**f**). Acetylated proteins in **f** were quantified (**g**). **h**, NAD^+^ levels in *eater*^*1*^ and *CantonS* heads. **i**,**j**, Sleep analysis in female wild-type and *eater* mutant with or without nicotinamide supplementation. The graph represents sleep in *CantonS* and *eater*^*1*^ (**i**). Sleep was quantified in genotypes shown (**j**); *n* = 31, 32, 30 and 30 from left to right. **k**,**l**, ROS in the brain. *eater*^*1*^ has more MitoSox incorporation than *CantonS* (**k**). The MitoSox signal was quantified (**l**); *n* = 17 for *CantonS* and 19 for *eater*^*1*^. NS, *P* > 0.01); *P < 0.1; **P < 0.01, ***P < 0.001, ****P < 0.0001. Horizontal bars in graphs, median; error bars in **i**, s.e.m. White and black bars below the graph in **i** represent day and night, respectively. *n* represents biologically independent samples. Two-sided Mann–Whitney test was performed for data analysis in **b**, **d**, **e**, **i** and **l**. Two-sided unpaired *t*-test was performed for **g** and **h**. Detailed statistics in Supplementary Table 1. Scale bars, 10 μm (**a**); 100 μm (**k**). a.u., arbitrary unit. IB, immunoblot; IP, immunoprecipitation; LysAC, acetylated lysine; Tub, α-tubulin.

In previous in vitro experiments, domains 1–199 of the Eater protein were found to interact with acetylated LDL or oxidized LDL^30^. To test the affinity of haemocyte-expressed Eater for acetylated or oxidized LDL versus neutral LDL, we used an ex vivo system where wild-type or *eater* mutant haemocytes were cultured with neutral, acetylated or oxidized LDL. Consistent with previous studies^30^, neither wild-type nor *eater* mutant haemocytes exhibited any affinity for neutral LDL (Extended Data Fig. 5d,e). However, wild-type haemocytes bound more oxidized (Extended Data Fig. 5f,g) and acetylated (Fig. 5a,b) LDL than *eater* mutants. Most of the acetylated LDL remained outside the haemocyte (Fig. 5a,b), whereas oxidized LDL was observed intracellularly (Extended Data Fig. 5f,g). This suggests that haemocytes have different affinities or uptake properties for these modified forms of LDL. Oxidized LDL is taken up predominantly by the CD36 homologue *croquemort* (Crq) in *Drosophila*^26^, but knockdown of *crq* in Hml^+^ haemocytes or *crq* mutants did not show a strong sleep phenotype (Extended Data Figs. 2a and 5h). On the other hand, haemocyte Eater affects sleep and affects uptake of both oxidized and acetylated lipids.

We considered the possibility that acetylated lipoproteins, rather than acetylated lipids per se, are targeted by Eater. LD transfer is mediated typically by apolipoproteins^41^, one such being GLaz—the *Drosophila* orthologue of apolipoprotein E/D. GLaz is known to be acetylated and knockdown of GLaz decreases sleep in flies^11^. However, immunoprecipitation assays indicated that levels of acetylated GLaz are similar between wild-type flies and *eater* mutants (Extended Data Fig. 5i,j and Supplementary Fig. 1), suggesting that it does not contribute to the *eater* phenotype. We found that *eater* mutants exhibit an overall increase in acetylated proteins compared with wild-type (Fig. 5c,d and Supplementary Fig. 1).

## Eater is required for brain function and lifespan

Numerous proteins undergo acetylation, and this modification is conserved across a wide range of species, from nematodes to humans^42,43^. Acetylation regulates various cellular processes, including mitochondrial metabolism, protein translation, protein folding and DNA packaging^42^. It can be catalysed by enzymes such as histone acetyltransferases, or it can occur non-enzymatically when acetyl-CoA levels are elevated in the cell^42^.

Given the increase of acetylated proteins in *eater* mutants (Fig. 5c,d and Supplementary Fig. 1), we investigated whether the levels of acetyl-CoA were elevated compared with wild-type flies. Notably, the concentration of acetyl-CoA in *eater* mutants was more than twice that of wild-type flies (Fig. 5e). We sought to identify candidate proteins that may be targeted by the high acetyl-CoA, and so considered PGC1α and DRP1, which regulate mitochondrial biogenesis and mitochondrial fission, respectively, and whose acetylation affects proper mitochondrial activity^44,45^. To determine whether PGC1α or DRP1 is more acetylated in the *eater* mutant brain, we immunoprecipitated acetylated lysine and immunoblotted for the PGC1α and DRP1 proteins (Fig. 5f,g and Supplementary Fig. 1). We found that acetylation of both spargel (srl; *Drosophila* homologue of PGC1α) and DRP1 is increased in *eater* mutants (Fig. 5f,g and Supplementary Fig. 1). As compromised mitochondrial activity can affect NAD levels, we measured these in *eater* mutants and found that NAD^+^ and NADH levels were lower than in the controls (Fig. 5h and Extended Data Fig. 5k).

To determine whether reducing acetylation could rescue sleep in *eater* mutants, we overexpressed the deacetylase enzyme sirtuin^46^ in glia; however, this intervention did not restore total sleep in *eater* mutants, and instead reduced sleep by itself (Extended Data Fig. 5l). As sirtuin activity depends on NAD^+^, the depleted NAD^+^ levels in *eater* mutants may explain the lack of rescue by sirtuin overexpression. Indeed, supplementing fly food with 10 mM nicotinamide—a precursor for NAD synthesis that can restore NAD^+^ levels and thereby enhance endogenous deacetylase activity—partially rescued the *eater* sleep phenotype. Feeding nicotinamide had no effect on sleep in wild-type flies (Fig. 5i,j).

Given the acetylation of key mitochondrial proteins, we assessed mitochondrial integrity by measuring reactive oxygen species (ROS). As a first step, we visualized ROS in the brain using two different fluorescent probes: MitoSox and DHE^47^ (Extended Data Fig. 5m). Because we were particularly interested in cortex glia, we co-localized with a cortex glia marker and compared ROS levels between wild-type and *eater* mutant flies (Extended Data Fig. 5m); *eater* mutants exhibited higher ROS levels than wild-type flies (Fig. 5k,l), with no apparent cell death in the brain (Extended Data Fig. 5n). Altogether, our results indicate that when lipids are not cleared from glial cells by haemocytes, the resulting lipid accumulation in glia leads to metabolic stress, including increased acetyl-CoA, reduced NAD^+^, mitochondrial dysfunction caused by DRP1 or PGC1α acetylation and elevated ROS levels. This metabolic stress probably contributes to sleep loss in *eater* mutant flies.

**Fig. 6:**
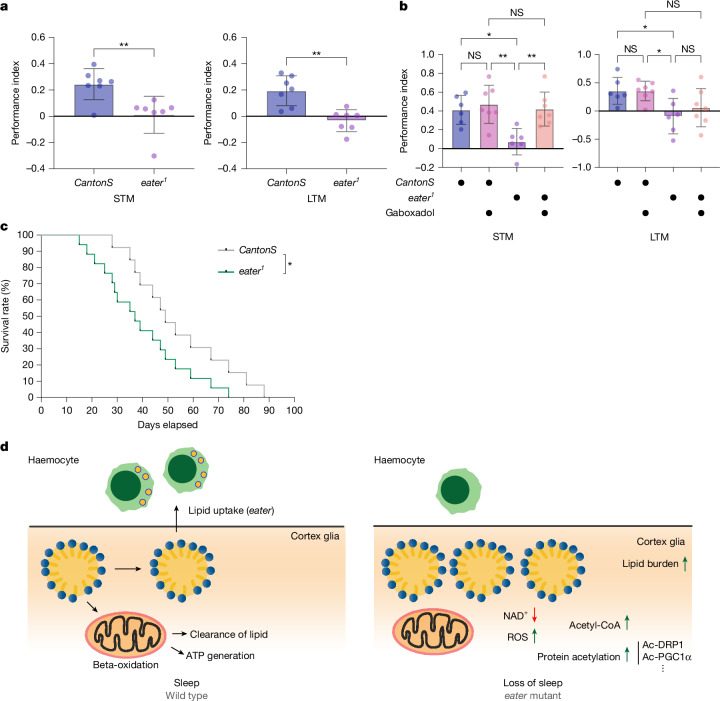
*eater* mutants have memory deficits and reduced lifespan. **a**,**b**, Measurement of short-term memory (STM) or long-term memory (LTM) in wild-type (*CantonS*; *n* = 7) or *eater* mutants (*eater*^*1*^; *n* = 7). *eater*^*1*^ exhibit impairments in both short-term memory and long-term memory (**a**). Gaboxadol treatment of *eater*^*1*^ for 2 days (**b**) rescues short-term memory defects (left) but not long-term memory (right); *n* = 6, 6, 7 and 7 from left to right. **c**, Comparison of lifespan between *CantonS* and *eater*^*1*^ flies. *eater* mutants showed reduced lifespan relative to wild type; *n* = 300 of each. **d**, Schematic illustration of haemocyte–glia interaction during sleep. LDs can be eliminated in two distinct pathways: one involves lipid catabolism through beta oxidation in the cortex glia, the other involves the uptake of LDs from the cortex glia by haemocytes through Eater. When haemocyte-mediated lipid uptake is disrupted, LDs accumulate in cortex glia, leading to increased protein acetylation and ROS levels while reducing NAD^+^ levels. This metabolic regulation between glia and haemocytes is crucial for maintaining proper brain lipid metabolism. Sleep promotes this metabolic regulation and is reduced when the process is disrupted. Two-sided unpaired *t*-test (**a**), two-sided Tukey’s multiple comparisons test (**b**) or log-rank (Mantel–Cox) test (**c**) were performed for data analysis. *n* are based on biologically independent experiments. Each dot represents more than 100 flies per experiment. Bars in graphs: the mean with s.d. Images were generated by Illustrator. NS, *P* > 0.01; *P < 0.1; **P < 0.01, ***P < 0.001, ****P < 0.0001. Detailed statistics in Supplementary Table 1.

The reduced sleep and dysregulated metabolic processes in *eater* mutants led us to ask whether memory and lifespan were affected. As seen in some other sleep mutants^48,49^, *eater* mutant flies exhibited deficits in both short-term and long-term memory (Fig. 6a,b) and shorter lifespan compared with wild-type flies (Fig. 6c). To determine whether memory loss in *eater* mutants derives from reduced sleep, we treated flies with gaboxadol for 2 days before the memory experiment. Gaboxadol feeding had no effect on wild-type flies, but in *eater* mutant flies it was enough to rescue short-term memory but not long-term memory (Fig. 6b).

## Discussion

We show here that haemocytes are recruited to the brain during periods of increased sleep and that they clear lipids by means of the Eater protein. If Eater function is impaired, glia accumulate more lipids, and the lipid burden induces metabolic stress with an increase in protein acetylation. This causes mitochondrial dysfunction and metabolic imbalance in the brain (Fig. 6d). In addition to disrupted sleep, memory is impaired and lifespan is shortened. These findings highlight a critical role of brain–periphery interaction, specifically glia–haemocyte lipid transfer, in maintaining brain metabolic health during sleep.

Most studies of immune–sleep interaction have focused on active immune states like inflammation, specific disease or sleep deprived conditions^7^. In our study we aimed to investigate the interaction between the immune system and sleep in normal daily conditions, where the immune system is not active. We focused on the role of immune cells. To achieve this, we used the simple model organism *Drosophila* and found that macrophage-like immune cells—haemocytes—in the circulation track to the head during sleep. Notably, these haemocytes are quite localized in the brain, and are not found in the ventral nerve cord, perhaps because they require specialized regions of the BBB. Peak recruitment is during times of high sleep, with head recruitment falling off later in the night. This may indicate that sleep homeostatic drive has been discharged, and is seen with other sleep-dependent processes—for example, endocytosis through the BBB and proboscis extension, which start to decline even earlier in the night^50,51^. It is also possible that haemocyte recruitment to the brain occurs during specific stages of sleep, which have now been described in the fly.

Screening genes expressed in haemocytes for effects on sleep identified the gene *eater*, which encodes a protein with 32 EGF-like repeats that is involved in cell–cell adhesion, LDL uptake and phagocytosis of Gram-positive bacteria^30^. *eater* mutant flies show reduced total sleep and increased sleep fragmentation (Fig. 2a,b) along with memory defects and reduced lifespan (Fig. 6a–c). We were able to completely rescue sleep as well as localization and lipid uptake phenotypes of *eater* mutants by expression of *eater* in haemocytes (Fig. 2c,d).

We find that haemocytes take up lipids from brain cortex glia by the Eater receptor. The lipids taken up are probably those that are transferred to cortex glia from neurons to prevent wake-induced damage to neuronal mitochondria^11^. Thus, the whole process relieves oxidative burden on the brain, which is supported by the increased oxidation seen in the absence of Eater. Although transporters that mediate neuron–glia transfer have been identified^11^, how exactly lipids are transferred from cortex glia to haemocytes is unclear. Our data confirm physical contact between haemocytes and glial cells, supporting direct haemocyte–glia interactions. Given the direct contacts we see between BBB glia and haemocytes, we speculate that LDs from cortex glia are transferred through the BBB to haemocytes; direct contacts between haemocytes and cortex glia may also occur, but would require validation. Although LD accumulation occurs predominantly in cortex glia, we note that other glial subpopulations, in particular astrocytes, also transfer LSD2::GFP-labelled lipids to haemocytes. It is possible that astrocytes process lipids without accumulating LDs or, alternatively, that they transfer droplets or fatty acids through lipoprotein particles to other glia^37^. Ultimately, many of these lipids end up in haemocytes. However, some are probably also processed in glia by beta-oxidation, thereby generating energy. Likewise, we speculate that the lipids transported in haemocytes are processed, either within the haemocytes themselves or in the fat body. Specific lipid binding/processing molecules have been implicated in the regulation of sleep^52^.

In addition to increased LDs, *eater* mutant brains had higher levels of acetylated proteins, increased acetyl-CoA levels and reduced NAD^+^ levels. Although the sequence of these changes remains uncertain, we propose that loss of lipid uptake by Eater leads to an accumulation of LDs, triggering metabolic stress characterized by elevated acetyl-CoA, increased acetylation of key mitochondrial proteins and impaired mitochondrial function. Increased acetyl-CoA levels are also indicative of less beta-oxidation and lower energy production. This fuels a vicious cycle of metabolic stress, oxidative damage and LD accumulation. The consequently reduced levels of NAD^+^ may further contribute to increased acetylation by impairing NAD-dependent deacetylase enzymes, such as sirtuins^46^. However, we cannot exclude the possibility that increased acetyl-CoA levels are a consequence of reduced uptake of acetylated lipids by Eater. Alternatively, lower NAD may be an early outcome of metabolic stress and, by reducing activity of deacetylases, could account for higher acetylation of DRP1 and PGC1α. Acetylation of PGC1α is known to inhibit its function^44^ whereas acetylation of DRP1 increases its activity but induces metabolic stress and cellular dysfunction^45^. Haynes et al. previously demonstrated that knockdown of *Drp1* in neurons or glia decreases sleep, as does knockdown of beta-oxidation-related genes, such as *Mcad*^11^. Similar impairments of mitochondrial function probably result from acetylation of DRP1 and PGC1α, the latter being a transcription factor that promotes mitochondrial biogenesis and the expression of beta-oxidation-related genes^45^.

Protein acetylation (beyond histones) has been studied mostly in the context of metabolic syndromes such as alcoholic liver disease, high fat diet or atherosclerosis^53^, with less known about its role in other biological processes. We found that *eater* mutants have elevated acetylated proteins and lower NAD^+^ levels. Recent research on short sleep mutants has identified decreased NAD^+^ levels in the brain^54^ but, to our knowledge, protein acetylation has not been examined in the context of sleep regulation. These findings, including the reduced sleep produced by SIRT1 overexpression, suggest that the interplay between NAD^+^ levels and protein acetylation in the brain may have a critical role in sleep control and function.

Although brain–periphery interactions are currently receiving attention, the role we report here for haemocytes is unprecedented. Our findings suggest that oxidated and acetylated lipids need to be removed from the brain by haemocytes to prevent oxidative damage and preserve the integrity of brain mitochondria. In mammals, microglia are key glial cell types that take up lipids from neurons, and are particularly important in the context of neurodegeneration^55^. As *Drosophila* lack microglia, circulating haemocytes may serve an analogous function, acting as intermediaries for lipid uptake and transport/storage and combating stress by accumulating LDs. We find that this is a sleep-dependent process. Although sleep is thought to promote clearance in the brain, the idea that peripheral blood cells contribute to this process represents a critical new perspective.

## Methods

### *Drosophila* strains and fly husbandry

All flies for experiments were maintained at 25 °C in a 12 h–12 h light–dark cycle, except for the temperature-sensitive Gal80 experiment in which Gal80 flies were kept at 18 °C until the experiment and activated Gal4 at 30 °C for 2 days. For the gene switch experiment, 500 μM mifepristone (RU486, sigma, catalogue no. M8046) was added to sucrose/agar food. For the RU486 control food, the same amount of 80% ethanol was added to sucrose/agar food. The following *Drosophila* stocks were used in this study: CantonS (Sehgal laboratory stock), Hml*Δ*-LexA LexAop-mCherry (J. Shim), Hml*Δ*-Gal4 UAS-EGFP (BL30139, BL30140), Srp-Hemo-mCherry (BL78358, BL78359, BL78362, BL78363), 9-137-Gal4 (Sehgal laboratory stock), NP2222-Gal4 (Sehgal laboratory stock), C584-Gal4 (Sehgal laboratory stock), TH-Gal4 (BL8848), R23E10-Gal4 (BL49032), R85G01-LexA (BL54285), R54C07-LexA (BL61562), Srp-Gal4 (L. Waltzer), Srp-Hemo-Gal4^DBD^ (I. Evans), Srp-Hemo-Gal4^AD^ (I. Evans), eater-dsRed (U. Banerjee), Ppn-Gal4 (BL77733), NP6293-Gal4 (Sehgal laboratory stock), moody-Gal4 (Sehgal laboratory stock), Repo-GeneSwitch (Sehgal laboratory stock), UAS-CD4::GRASP (BL58755), eater^1^ (BL68388), UAS-eater (BL36325), eater RNAi (BL25863, V4301), UAS-mCD8GFP (BL5137), UAS-mCD8::RFP, LexAop-mCD8::GFP (BL58754) UAS-LD::GFP (M. Welte), UAS-GFP::LSD2 (M. Welte), UAS-Sirt1 (BL44216), GLaz-GFSTF (BL60526), UAS-h.MEGF11(BL78460), MZ0709-Gal4 (M. Freeman), Alrm-Gal4 (M. Freeman), Repo-Gal4 (L. Griffith), Repo-LexA (M. Freeman), put RNAi (BL35195), Lsd-1 RNAi (V30884), NimC3 RNAi (V22920), GLaz RNAi (V4806), Lsd-2 RNAi (V40734), UAS-PvrDN (BL58431), Nplp2 RNAi (BL54041), NimC1 RNAi (BL25787), NimC4 RNAi (BL61866), NimB1 RNAi (BL55937), NimC2 RNAi (BL25960), Smox RNAi (BL41670), PGRP-LC RNAi (BL33383), drpr RNAi (BL67034), NimB4 RNAi (BL55963), Apoltp RNAi (BL 51937), NimA RNAi (V104204), Col4a1 RNAi (BL44520), NimB2 RNAi (BL62289), NimB5 RNAi (V15758), babo RNAi (BL25933), Karl RNAi (V9446), LpR2 RNAi (BL31150), apolpp RNAi (BL28946), Acsl RNAi (V3222), LpR1 RNAi (BL27249), crq RNAi (BL40831), NimB3 RNAi (V330502).

### Sleep recording

For the fly sleep recording, mated 5- to 7-day-old flies were loaded into glass tubes containing 5% sucrose with 2% agarose. At least 2 days after loading into the monitors, sleep was analysed for 3  days. Single beam monitors were used for RNAi screening and the sleep deprivation experiment, but other sleep recordings were performed with multibeam monitors. Sleep was defined as failure of the fly to cross the red beam in the monitor for 5 or more minutes, analysis of data was performed with an in-house built code as described previously^56^.

### Sleep deprivation

We used mechanical sleep deprivation. To achieve deprivation, flies in single beam monitors were fixed to a vortex machine and shaken randomly for 2 s every 20 s over a 12-h period (from ZT12–ZT24). To estimate rebound sleep, 6 h of sleep after sleep deprivation was compared with sleep on the pre-deprivation day at the same ZT time (ZT0–ZT6). Analysis of per cent sleep gain was as described previously^57^. In short, 6 h of daytime sleep on the day before deprivation was subtracted from the 6 h after deprivation (sleep gain). Similarly, sleep loss was calculated by subtracting sleep during deprivation to night-time sleep a day before the deprivation (sleep loss). Percentage of sleep gain was calculated by amount of sleep gain relative to amount of sleep loss.

### Fly tissue clearing and haemocyte quantification

For visualizing circulating haemocytes, we optimized two different protocols^58,59^. Heads of female *HmlΔ-LexA LexAop-mCheery* flies were cut with a micro-scissor and fixed with 4% paraformaldehyde for 4 h at room temperature with rotation. After fixation, heads were incubated with 100% methanol at 4 °C overnight. Methanol was removed the following day and heads were incubated with BABB solution (2:1 ratio of benzyl benzoate and benzyl alcohol) for at least 6 h. After removing the BABB solution, heads were mounted on glass slides with VECTASHIELD solution without 4′,6-diamidino-2-phenylindole (DAPI). Imaging was performed immediately after mounting and a 408-nm excitation laser was used for auto-fluorescent signals. For thermogenetic experiments where wake or sleep neuronal populations were manipulated, flies were maintained at 18 °C until TrpA1 was activated for 1 day at 30 °C. Following this, they were processed as above. For haemocyte counts, we used the three-dimensional (3D) object counter in ImageJ software.

### Immunohistochemistry

Brains were dissected from female flies, fixed in a 4% paraformaldehyde solution and washed three times using 0.4% PBS TritonX-100. After three washes, samples were blocked using 10% normal goat serum for 30 min at 25 °C. Samples were incubated with the desired primary antibodies overnight at 4 °C and then washed three times using 0.4% PBS TritonX-100 (PBST). Samples were incubated with secondary antibodies (Life Tech, catalogue nos. A32723, A32740, A32742, A32731 and A21236) diluted 1:250 for 2 h. Samples were then washed three times with 0.4% PBST. After washing, samples were rinsed and kept in VECTASHIELD until they were mounted on glass slides. For haemocytes count, we did not remove air sacs to maximize the number of haemocytes. The following primary antibodies were used: α-NimC1 (a gift from I. Ando; 1:100), α-brp (Developmental Studies Hybridoma Bank (DSHB), catalogue no. nc82; 1:100), α-Repo (DSHB, catalogue no. 8D12; 1:100), α-cleaved dcp1 (Cell Signaling, catalogue no. 9578S; 1:100), Oil-Red O (Sigma, catalogue no. O9755) and BODIPY 493/503 (Fisher, catalogue no. D3922, 1:1,000). Images were obtained with a Leica Stellaris STED confocal microscope. For haemocyte counts, we used the 3D object counter in ImageJ software.

### Staining of LDs using Oil-Red O

Brains from female flies were dissected and fixed as for immunohistochemistry. After fixation, brains were washed three times with 0.4% PBS TritonX-100 and kept in 0.4% PBST overnight at 4 °C. If a sample needed primary antibody incubation, it was blocked with 10% normal goat serum in 0.4% PBS TritonX-100 for 30 min and then kept in diluted antibody with 0.4% PBST overnight at 4 °C. The following day, Oil-Red O solution (0.1 g per 20 ml of isopropanol; Sigma, catalogue no. O0625) was prepared in 0.4% PBST as a 2:3 ratio. If not stained with primary antibody, the sample was incubated with Oil-Red O solution for 10 min and washed with distilled water five times for 5 min each. If samples were stained with primary antibody, samples were washed and treated with secondary antibody and, after the secondary antibody, incubated with Oil-Red O solution. Finally, samples were rinsed with PBS and mounted in VECTASHIELD until they were mounted on glass slides. Images were obtained with the Leica Stellaris STED confocal microscope.

### Quantification of LDs in the brain

To count LDs, images were analysed with a custom ImageJ macro. For each slice in the stack, the BioVoxxel toolbox was used to subtract background noise using the convoluted background subtraction method with a mean convolution filter of 3 radius. Once the background was subtracted, the image was duplicated, and one of the copies was converted into a mask that contained only the brain region. Inside the masked area, LDs were counted using the Analyze Particles tool, defining a particle size of 2–250 and circularity of 0.4–1.0. Reported results are the sum of particles in the whole stack. The macro is publicly available at https://github.com/CamiloGuevaraEsp/lipid_droplets.

### Immunoprecipitation and western blot

In a 15-ml tube, at least 100 mixed-sex flies were collected for immunoprecipitation, or 20 flies for western blot. Flies were frozen on dry ice for 10 min. After freezing, flies were vortexed for 10–20 s three times to shake off heads. Flies were then poured into a sieve that allows passage only of heads. Fly heads were homogenized at 25 Hz for 2 min in a TissueLyser II (Qiagen) in 100 μl of lysis buffer for immunoprecipitation (250 mM Tris-HCl (pH 7.5), 250 mM NaCl, 1.5 M sucrose, 1% TritonX-100, protease inhibitor cocktail) or for western blot (RIPA buffer, Lifetech, catalogue no. 89901) with a 5-mm stainless steel bead (Qiagen, catalogue no. 69989) in round-bottom tubes (USA Scientific, catalogue no. 1620-2700). Homogenized samples were transferred to 1.7-ml microcentrifuge tubes and spun at 14,000 rpm for 10 min at 4 °C. Supernatants were used for the experiment. For immunoprecipitation, protein A/G magnetic agarose beads (Fisher, catalogue no. 78609) were used for antibody conjugation. Antibody conjugation to the bead or incubation of antibody with samples was performed in the cold room overnight. Samples were run in a 4–12% premade gel (Life Tech, catalogue no. NP0322). The following antibodies were used: anti-acetylated lysine-mouse (Life Tech, catalogue no. MA12021; 1:1,000), anti-acetylated lysine-rabbit (Cell Signaling, catalogue no. 9441S; 1:1,000), anti-DRP1 (a gift from L. Fisher; 1:1,000), anti-SRL (a gift from A. Duttaroy, 1:1,000), anti-α-tubulin (DSHB, catalogue no. 12G10; 1:1,000), anti-FLAG (Sigma, catalogue no. F3165; 1:2,000), anti-mouse-horseradish peroxidase (HRP) (Jackson Immuno, catalogue no. 715-035-151; 1:2,000), anti-rabbit-HRP (Jackson Immuno, catalogue no. 715-035-152; 1:2,000) and HRP signal was obtained with ECL substrate (Life Tech, catalogue no. 32209).

### Flow cytometry

At least 100 *HmlΔ-Gal4 UAS-EGFP* fly heads were cut with a micro-scissor under the microscope and kept in a round-bottom tube with 1,000 μl of ice-cold Schneider’s medium (Life Tech, catalogue no. 21720024) until the cutting was finished. With a metal bead, fly heads were homogenized with a TissueLyser II (Qiagen) at 25 Hz for 2 min. Samples were centrifuged at 6,000 rpm, for 5 min at 4 °C. Supernatant was discarded and pellets were treated with 37 °C pre-warmed 100 μl of Collagenase Type C (100 mg ml^−1^, Worthington-Biochem, catalogue no. LS004140), 380 μl of PBS, 20 μl of Dispase II (100 mg ml^−1^, Sigma, catalogue no. D4693). Samples were incubated on a rotator for 15 min and pipetted with a 200 μl pipet every 5 min. Then, 200 μl of ice-cold PBS was added to the sample, which was transferred to a 1.7-ml microcentrifuge tube. Samples were spun at 6,000 rpm, for 5 min at 4 °C. The supernatant was discarded and pellets were resuspended in 500 μl of cold Schneider’s medium; 0.5 μl DAPI (1 mg ml^−1^) was added and, after a short vortexing, samples were spun at 6,000 rpm for 5 min at 4 °C. Again, supernatant was discarded, and samples were resuspended in 600 μl of Schneider’s medium. Debris or clumps were removed using a 40-μm strainer (Sigma, catalogue no. BAH136800040) and samples were transferred to a 5-ml fluorescence-activated cell sorting (FACS) sorting tube. We used an Aria FACS sorter (BD Biosciences) with a 100-μm nozzle. Usually, 100 fly heads yield approximately 400 GFP^+^ haemocytes after sorting. The detailed cell gating strategy is in Supplementary Fig. 1.

### Haemocyte transfer

At least 30 larvae were dissected in Schneider’s medium (Life Tech, catalogue no. 21720024) and kept on ice during the dissection. Samples were spun at 6,000 rpm for 5 min at 4 °C. Haemocytes were resuspended with 100 μl of PBS for a cell density of 100–150 cells per microlitre. Using the microneedle, 2–3 μl of haemocytes were injected into the fly thorax. *HmlΔ-LexA LexAop-mCherry* haemocytes were used for validating haemocyte transfer.

### In vitro haemocyte culture

Larvae were dissected in 15 μl of Schneider’s medium (Life Tech, catalogue no. 21720024). Haemocytes were transferred to Schneider’s medium containing Dil-labelled neutral, oxidized or acetylated LDL (1:100 dilution, Life Tech, catalogue nos. L3482, L34358, L3484) in the tube. Haemocytes were incubated on Teflon printed microscopic slides (Immune-Cell, catalogue no. 61-100-17) for 2 h in the cold room. After 2 h, haemocytes were fixed with 4% paraformaldehyde and washed three times with 0.4% PBST. Haemocytes were kept in VECTASHIELD with DAPI before the imaging; images were obtained with a Leica Stellaris STED confocal microscope.

### NAM food feeding

Flies were raised on normal food for 5 days after eclosion and then transferred to sleep recording glass tubes that contain 5% sucrose and 2% agarose food with or without 10 mM nicotinamide (Sigma, catalogue no. 72345).

### Gaboxadol feeding

Gaboxadol hydrochloride (Sigma, catalogue no. T101) was dissolved in the normal fly food at a 2 mM concentration. Flies were kept in this for 2 days.

### Acetyl-CoA measurement

To measure acetyl-CoA in the fly head, ten female flies were collected in the 15-ml falcon tube and frozen on dry ice for 10 min. After freezing, flies were vortexed for 10–20 s three times to shake off heads. Flies were then poured into a sieve that allows passage only of heads. Ten fly heads were homogenized at 25 Hz for 2 min in a TissueLyser II (Qiagen) in extraction buffer from the acetyl-CoA colorimetric assay kit (Elabscience, catalogue no. E-BC-K652-M). After homogenization, all procedures followed the manufacturer’s instructions from the kit.

### NAD^+^/NADH measurement

To measure NAD^+^/NADH in the fly head, ten male and female *CantonS* and *eater*^*1*^ mutant flies were collected around ZT6 in 1.5-ml tubes then flash-frozen on dry ice. After freezing, flies were vortexed, and ten heads were collected then placed in a 2-ml tube with metal beads and 1 ml of lysis buffer (1:1 PBS: extraction buffer (10% DTAB 0.2 M NaOH)). The heads were homogenized using a TissueLyser II (Qiagen) at 25 Hz for 2 min. The homogenized liquid was passed through homogenizer tubes (Invitrogen, catalogue no. 12183-026) in a centrifuge for 5 min at 15,000 rpm at 4 °C. To measure NAD^+^ and NADH individually, 200 μl of lysate was added to separate 1.5-ml tubes; 100 ml of 4 M HCl was added to the NAD^+^ tube, and both were heated for 15 min at 60 °C. After incubation at room temperature for 10 min, 100 μl of 0.5 M Trizma base buffer was added to the NAD^+^ tube, and 200 μl neutralization buffer (a 1:1 mixture of 0.5 M Trizma: 0.4 M HCl) was added to the NADH tube. NAD^+^ samples were diluted 1:5 and NADH samples were diluted 1:1 using dilution buffer. Samples and standards were prepared 1:1 with NAD^+^/NADH-Glo detection reagent (Promega, catalogue no. G9071) according to manufacturer’s instructions, seeded onto a 384-well plate, and measured using a BioTek Cytation 5 imaging reader and the accompanying Gen5 v.3.12 software. Individual data points are the mean of three technical replicates.

### MitoSox staining

To stain the fly brain with MitoSox (Fisher, catalogue no. M36008), ten fly brains were dissected in Schneider’s medium and kept in medium until the dissection was finished. Brains were transferred to Schneider’s medium with MitoSox dye (final concentration 5 μM) and incubated for 10 min with rotation at room temperature. After the incubation, brains were washed with Schneider’s medium three times for 3 min with rotation. Brains were fixed with 4% paraformaldehyde for 2 min and rinsed with PBS. MitoSox signal was imaged immediately after mounting with VECTA SHIELD using a Leica Stellaris STED confocal microscope.

### Memory experiment

The memory experiment was as published^60^. In brief, 100 flies, 3–6 days old and of mixed sex, from the same genotype, were starved in agarose for 18 h. On the following day, flies were trained at 25 °C in 70% humidity in a small chamber containing 1.5 M sucrose or water soaked Whatman paper with odours (1:200 ratio of 4-methylcyclohexanol or 1:80 ratio of 3-octanol in paraffin oil) for 2 min under red light. After training, flies were placed in the bidirectional choice apparatus, which has an odour in each end. To remove bias coming from the odour, appetitive training was performed reciprocally. For long-term memory, trained flies were kept again in the agarose food for 18 h and the memory experiment was performed without re-training.

### Lifespan experiment

Three hundred age-matched female or male flies were collected for 5 days and transferred to vials of 30 flies each. Every 2 or 3 days, flies were flipped into new vials and the number of flies was counted.

### Haemocyte sample preparation for mass spectrometry analysis

From the *HmlΔ-Gal4 UAS-EGFP* fly, haemocytes were sorted by flow cytometry and 4,000 GFP^+^ cells were sorted into a 1.7-ml microcentrifuge tube. GFP^+^ cells were centrifuged at 6,000 rpm for 5 min at 4 °C, and pellets were kept at −80 °C until lipid extraction. Lipid extraction from fly haemocytes was performed using a modified Bligh and Dyer method^61^. In brief, frozen cell pellets were thawed at room temperature for 10 min before the addition of 200 µl of ultrapure water to facilitate cell lysis. This was followed by the addition of 450 µl of methanol and 250 µl of HPLC-grade chloroform. During extraction, 5 µl of 1 µg ml^−1^ of an internal standard mixture comprised of equiSPLASH (Avanti Polar Lipids, catalogue no. 330731), fatty acid 16:0-d2 (Cayman Chemical) and carnitine 14:0-d3 (Cayman Chemical) was added to each sample. The samples were vortexed for 10 s to form a single-phase solution and incubated at 4 °C for 15 min. Subsequently, 250 µl of ultrapure water and 250 µl of chloroform were added, inducing phase separation. The samples were then centrifuged at 16,000*g* for 10 min. The organic phase, containing the extracted lipids, was transferred carefully to fresh tubes and evaporated using a vacuum concentrator to obtain dried lipid extracts.

The dried lipid extracts were reconstituted in 200 µl of a 3:1 methanol:chloroform (MeOH:CHCl_3_) mix containing 10 mM ammonium formate. Following reconstitution, all samples were analysed using MRM methods. An injection solvent containing 0.02 µg ml^−1^ EquiSPLASH (Avanti Polar Lipids, catalogue no. 330731) was used as a quality control sample to monitor peak stability over time.

### Unbiased lipidomics using MRM-profiling

Lipidomic analyses were performed using an Agilent 6495C triple quadrupole mass spectrometer coupled to an Agilent 1290 Infinity II LC system with a G7167B autosampler. Samples were introduced into the Agilent Jet Stream (AJS) ion source by direct flow injection (no chromatographic separation). Mass spectrometry data were acquired for 3 min per injection. For each MRM scan, 8 μl of sample was injected. MRM methods were organized into 25 methods on the basis of the ten main lipid classes based on the LipidMaps database, spanning over a total of 3,000 individual lipid species. Triacylglycerols and diacylglycerols were divided into separate methods based on fatty acid neutral loss residues. All MRM data were processed using CLAW MRM^36^.

### Reporting summary

Further information on research design is available in the Nature Portfolio Reporting Summary linked to this article.

## Online content

Any methods, additional references, Nature Portfolio reporting summaries, source data, extended data, supplementary information, acknowledgements, peer review information; details of author contributions and competing interests; and statements of data and code availability are available at 10.1038/s41586-025-10050-w.

## Supplementary information

Supplementary Figure Raw, uncropped data for gels in the study and gating strategy of FACS for this study.

Reporting Summary

Supplementary Table This table is related to statistical details in Figs. 1–6 and Extended Data Figs. 1–5. Each tab indicates a figure.

## Data Availability

All data generated during and/or analysed in this study are included in this published Article and its Supplementary Information and materials that were newly generated for this study, such as plasmids and fly lines, are available from the corresponding author upon request. The lipidomics MRM data, along with the code for processing and visualization, are available at: https://github.com/chopralab/Sleep-dependent-clearance-of-brain-lipids-by-peripheral-blood-cells.
