# Supplementary material for: Sleep-dependent clearance of brain lipids by peripheral blood cells
Source: Nature. 2026 Feb 11;651(8106):720–31. doi: 10.1038/s41586-025-10050-w (PMC12999507; doi:10.1038/s41586-025-10050-w)
Supplement: Supplementary file 1 — Raw, uncropped data for gels in the study and gating strategy of FACS for this study. [file 41586_2025_10050_MOESM1_ESM.pdf]

nature portfolio

https://doi.org/10.1038/s41586-025-10050-w

Supplementary information

Sleep-dependent clearance of brain lipids by peripheral blood cells

In the format provided by the authors and unedited

a Figure 5.c

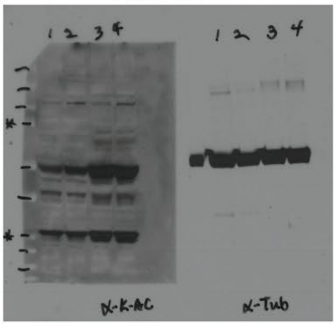

b Figure 5.f

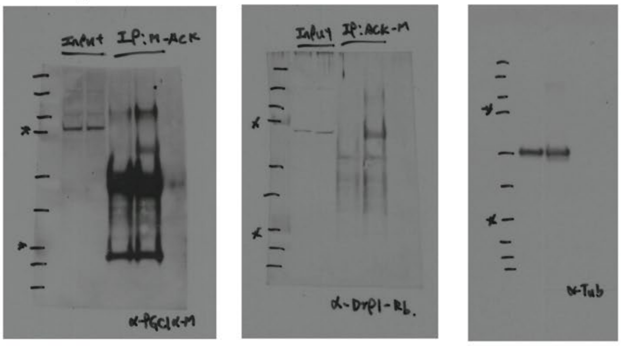

c Extended Data Figure 5.i

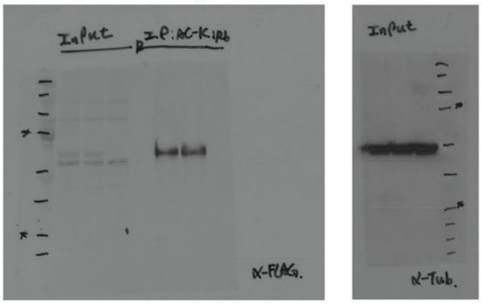

d

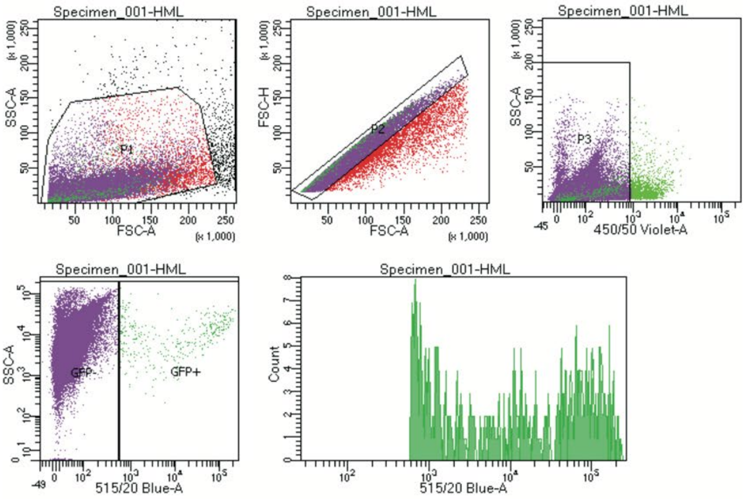

Tube: HML

Population	#Events	%Parent	%Total
All Events	100,000	####	100.0
P1	96,771	96.8	96.8
P2	93,015	96.1	93.0
P3	91,571	98.4	91.6
GFP-	91,208	99.6	91.2
GFP+	356	0.4	0.4

Supplementary Fig. 1 Raw Data for western blot and FACS gating strategy.

a-c. Uncropped western blot image from Fig. 5c,f and Extended Data Fig. 5i. Lanes 1,2 indicate head lysates from wild-type (*CantonS*) and 3,4 indicate *eater* mutant (*eater*^{1}). Left: western blot against acetylated lysine, right: western blot against alpha-tubulin **(a)**. Immunopreciptation of head lysates, using acetylated lysine antibody, from wild-type (*CantonS*) and *eater* mutant (*eater*^{1}). Input means protein lysates before the immunoprecipitation. Western blot was performed using PGC1α antibody (left) or DRP1 antibody (middle); anti alpha tubulin antibody was used for loading control (right). **(b)**. Immunopreciptation of head lysates, using acetylated lysine antibody, from wild-type (*CantonS*) and *eater* mutant (*eater*^{1}). Western blot was performed using FLAG antibody (left) and alpha tubulin was used for loading control (right). **(c)**. Asterisk at the bottom indicates a size of 25 kDa, and top indicates a size of 75 kDa. Detailed lane information is annotated in original figures.

d. GFP+ Hemocyte sorting from *HmlΔ*>GFP fly heads. P1 gating was set to capture most of the cell population excluding debris, while the P2 gating was set to select only single cell populations. Next, to isolate dead cells, only the DAPI negative population was collected in P3. Finally, GFP positive hemocytes with an intensity of 10^3 or higher were selected for our study. Wild-type (*CantonS*) fly heads were used as a negative control for appropriate GFP gating.
