# Supplementary material for: Sleep-dependent clearance of brain lipids by peripheral blood cells
Source: Nature. 2026 Feb 11;651(8106):720–31. doi: 10.1038/s41586-025-10050-w (PMC12999507; doi:10.1038/s41586-025-10050-w)
Supplement: Supplementary file 2 — Reporting Summary [file 41586_2025_10050_MOESM2_ESM.pdf]

nature portfolio

Corresponding author(s): Amita Sehgal

Last updated by author(s): Nov 13, 2025

Reporting Summary

Nature Portfolio wishes to improve the reproducibility of the work that we publish. This form provides structure for consistency and transparency in reporting. For further information on Nature Portfolio policies, see our Editorial Policies and the Editorial Policy Checklist.

Statistics

For all statistical analyses, confirm that the following items are present in the figure legend, table legend, main text, or Methods section.

n/a	Confirmed	
☐	☒	The exact sample size (*n*) for each experimental group/condition, given as a discrete number and unit of measurement
☐	☒	A statement on whether measurements were taken from distinct samples or whether the same sample was measured repeatedly
☐	☒	The statistical test(s) used AND whether they are one- or two-sided<br>*Only common tests should be described solely by name; describe more complex techniques in the Methods section.*
☐	☒	A description of all covariates tested
☐	☒	A description of any assumptions or corrections, such as tests of normality and adjustment for multiple comparisons
☐	☒	A full description of the statistical parameters including central tendency (e.g. means) or other basic estimates (e.g. regression coefficient) AND variation (e.g. standard deviation) or associated estimates of uncertainty (e.g. confidence intervals)
☐	☒	For null hypothesis testing, the test statistic (e.g. *F*, *t*, *r*) with confidence intervals, effect sizes, degrees of freedom and *P* value noted<br>*Give P values as exact values whenever suitable.*
☒	☐	For Bayesian analysis, information on the choice of priors and Markov chain Monte Carlo settings
☒	☐	For hierarchical and complex designs, identification of the appropriate level for tests and full reporting of outcomes
☐	☒	Estimates of effect sizes (e.g. Cohen's *d*, Pearson's *r*), indicating how they were calculated

Our web collection on statistics for biologists contains articles on many of the points above.

Software and code

Policy information about availability of computer code

Data collection	Sleep data was collected using a Drosophila Activity Monitoring (DAM) System (TriKinetics, Waltham, MA). Microscopic images were taken by Leica Stellaris STED confocal microscope.
Data analysis	All analysis codes used have been identified/cited and are publicly available. ImageJ/FIJI (Ver 1.54f): http://fiji.sc ; or Lipid analysis : https://academics.skidmore.edu/blogs/cvecsey/. For the sleep analysis, we used in-lab built code like previously described (https://academic.oup.com/sleep/article-lookup/doi/10.1093/sleep/zsaa265) with a use of MATLAB R2023b version. For the statistics, Prism version 10.6.1 (GraphPad, La Jolla, CA, USA) was used.

For manuscripts utilizing custom algorithms or software that are central to the research but not yet described in published literature, software must be made available to editors and reviewers. We strongly encourage code deposition in a community repository (e.g. GitHub). See the Nature Portfolio guidelines for submitting code & software for further information.

Data

Policy information about availability of data

All manuscripts must include a data availability statement. This statement should provide the following information, where applicable:

- Accession codes, unique identifiers, or web links for publicly available datasets
- A description of any restrictions on data availability
- For clinical datasets or third party data, please ensure that the statement adheres to our policy

All data generated during and/or analyzed in this study are included in this published article and its supplementary information and materials that were newly generated for this study, such as plasmids and fly lines, are available from the Lead Contact upon request.Hemocytes lipidomics data set have been deposited in https://github.com/chopralab/Sleep-dependent-clearance-of-brain-lipids-by-peripheral-blood-cells.

Research involving human participants, their data, or biological material

Policy information about studies with human participants or human data. See also policy information about sex, gender (identity/presentation), and sexual orientation and race, ethnicity and racism.

Reporting on sex and gender	human sources were not used in the experiment
Reporting on race, ethnicity, or other socially relevant groupings	human sources were not used in the experiment
Population characteristics	human sources were not used in the experiment
Recruitment	human sources were not used in the experiment
Ethics oversight	human sources were not used in the experiment

Note that full information on the approval of the study protocol must also be provided in the manuscript.

Field-specific reporting

Please select the one below that is the best fit for your research. If you are not sure, read the appropriate sections before making your selection.

☒ Life sciences ☐ Behavioural & social sciences ☐ Ecological, evolutionary & environmental sciences

For a reference copy of the document with all sections, see nature.com/documents/nr-reporting-summary-flat.pdf

Life sciences study design

All studies must disclose on these points even when the disclosure is negative.

Sample size	Sample sizes in each experiment were based on the number of samples that we were able to collect/dissect/process within 2hrs, while including all groups to be statistically compared to one-another. All the size informations were included in the manuscript.
Data exclusions	No data was excluded from the analyses. All attempts of replication were successful.
Replication	The experiments reported in this study were minimally performed triplicated.
Randomization	Collected flies were always randomly selected based on their genotype to perform all the experiments.
Blinding	Blinding was not used and was not relevant since all samples of all groups to be compared were always subjected to the same computer analysis programs with the same parameters.

Reporting for specific materials, systems and methods

We require information from authors about some types of materials, experimental systems and methods used in many studies. Here, indicate whether each material, system or method listed is relevant to your study. If you are not sure if a list item applies to your research, read the appropriate section before selecting a response.

Materials & experimental systems

n/a	Involved in the study
☐	☒ Antibodies
☒	☐ Eukaryotic cell lines
☒	☐ Palaeontology and archaeology
☐	☒ Animals and other organisms
☒	☐ Clinical data
☒	☐ Dual use research of concern
☒	☐ Plants

Methods

n/a	Involved in the study
☒	☐ ChIP-seq
☐	☒ Flow cytometry
☒	☐ MRI-based neuroimaging

Antibodies

Antibodies used	Primary antibody (In case of antibodies obtained from the non commercial way,relavant papers were represented on the validation section).<br>a-NimC1 (Gift from I. Ando, 1:100), a-brp (DHSB, nc82, 1:100), a-Repo (DSHB 8D12, 1:100), a-cleaved dcpl(Cell signaling, 9578S, 1:100), a-Acetylated lysine (Life Tech, MA12021 or Cell signaling, 9441S, 1:1000), a-DRP1(Gift from L.Fisher, 1:1000), a-SRL (Gift from A.Duttaroy, 1:1000), a-alpha Tubulin (DSHB, 12G10, 1:1000),a-FLAG (Sigma, F3165, 1:2000)<br>Secondary antibody: a-mouse-HRP (Jackson Immuno, 715-035-151, 1:2000),a-rabbit-HRP (Jackson Immuno, 715-035-152, 1:2000), fluorescent secondary antibodies (Life Tech, A32723, A32740, A32742, A32731, A21236).
Validation	Primary antibody:<br>NimC1 antibody was previously validated in previous paper (https://www.sciencedirect.com/science/article/pii/S0960982207010184).<br>brp (https://dshb.biology.uiowa.edu/nc82), alpha tubulin (https://dshb.biology.uiowa.edu/12G10-anti-alpha-tubulin) and repo (https://dshb.biology.uiowa.edu/8D12-anti-Repo) antibody was characterized and validated in various studies. More references were described in website.<br>DCP1 (1:100, rabbit; #9578, Cell Signaling) antibody was validate for immunohistochemistry (PMID: 27058168). More references in the manufacturer's website.<br>Acetylated lysine (Life Tech, MA12021 or Cell signaling, 9441S, 1:1000) antibody was validated for IP (PMID: 30886224 or PMID: 34348140). More references in the manufacturer's website.<br>DRP1 antibody was previously validated in previous paper (https://journals.plos.org/plosone/article?id=10.1371/journal.pone.0010054)<br>SRL antibody was previously validated in previous paper (https://www.sciencedirect.com/science/article/pii/S001216061830592X?via%3Dihub).<br>FLAG antibody antibody was validated for WB (PMID: 31417089). More references in the manufacturer's website.<br>References in the manufacturer's website for the secondary antibodies (https://www.jacksonimmuno.com/ or https://www.thermofisher.com/us/en/home/life-science/antibodies/secondary-antibodies/fluorescent-secondary-antibodies.html).

Animals and other research organisms

Policy information about studies involving animals; ARRIVE guidelines recommended for reporting animal research, and Sex and Gender in Research

Laboratory animals	Species : Drosophila melanogatser.<br>All fly lines used are publicly available and have been listed in the Material and Methods section. 7 -10day-old female or male flies were used for all experiments.
Wild animals	we did not used wild animals
Reporting on sex	We used both male and female flies and if presented one specific sex, we noticed on the manuscript.
Field-collected samples	samples we did not used field-collected smaples
Ethics oversight	Studies using Drosophila melanogaster are not subject to institutional ethical approval

Note that full information on the approval of the study protocol must also be provided in the manuscript.

Plants

Seed stocks	*Report on the source of all seed stocks or other plant material used. If applicable, state the seed stock centre and catalogue number. If plant specimens were collected from the field, describe the collection location, date and sampling procedures.*
Novel plant genotypes	*Describe the methods by which all novel plant genotypes were produced. This includes those generated by transgenic approaches, gene editing, chemical/radiation-based mutagenesis and hybridization. For transgenic lines, describe the transformation method, the number of independent lines analyzed and the generation upon which experiments were performed. For gene-edited lines, describe the editor used, the endogenous sequence targeted for editing, the targeting guide RNA sequence (if applicable) and how the editor was applied.*
Authentication	*Describe any authentication procedures for each seed stock used or novel genotype generated. Describe any experiments used to assess the effect of a mutation and, where applicable, how potential secondary effects (e.g. second site T-DNA insertions, mosiacism, off-target gene editing) were examined.*

Flow Cytometry

Plots

Confirm that:

☒ The axis labels state the marker and fluorochrome used (e.g. CD4-FITC).

☒ The axis scales are clearly visible. Include numbers along axes only for bottom left plot of group (a 'group' is an analysis of identical markers).

☒ All plots are contour plots with outliers or pseudocolor plots.

☒ A numerical value for number of cells or percentage (with statistics) is provided.

Methodology

Sample preparation	GFP+ Hemocytes from the fly head were used for experiments. All the detail method for sample preparation was described in the Method.
Instrument	Aria FACS sorter (BD Biosciences) with 100 µm nozzle
Software	We does not present any FACS data in the manuscript but for sorting we used instrument software.
Cell population abundance	Usually, 100 fly heads give approximately 400 GFP+ hemocytes after sorting.
Gating strategy	GFP+ DAPI- hemocytes live hemocytes were collected for future analysis. Experiments always done with GFP- DAPI- negative control samples.

☒ Tick this box to confirm that a figure exemplifying the gating strategy is provided in the Supplementary Information.
